# Influence of area-level social vulnerability on all-cause pneumonia incidence among adult Medicare and Medicaid enrollees

**DOI:** 10.1038/s43856-025-01163-4

**Published:** 2025-11-14

**Authors:** Salini Mohanty, Priya Shanmugam, Saumya Chatrath, Kelsie Cassell, Nicole Cossrow, Peter C. Fiduccia, Esther Smith-Howell, Valina C. McGuinn, Jelena Zurovac, Constance Delannoy, Alyssa Evans, Michael Barna, Aparna Keshaviah, Kristen A. Feemster

**Affiliations:** 1 Merck & Co., Inc, Rahway, NJ USA; 2 Mathematica Inc, Princeton, NJ USA

**Keywords:** Epidemiology, Public health

## Abstract

**Background:**

The incidence of pneumonia varies by demographic and clinical factors, but less is known about the influence of area-level social determinants of health.

**Methods:**

Using Medicare and Medicaid claims, we characterized the relationship between the county-level Minority Health Social Vulnerability Index (MHSVI) and all-cause pneumonia (ACP), pneumococcal pneumonia (PP) and invasive pneumococcal disease (IPD) incidence from 2016 to 2019.

**Results:**

We show that in Medicare ( ≥ 65 years, 55% female) and Medicaid (ages 19–64, 58% female), ACP incidence is 8052 and 1819 per 100,000 person-years, respectively. Across both cohorts, rates are highest among enrollees who are male, non-Hispanic white, older, at high risk for pneumococcal disease (those with immunocompromising or other serious conditions such as cancer), and rural county residents. Among high-risk Medicare and Medicaid enrollees, ACP incidence is higher in the most versus least socially vulnerable counties, whereas the opposite is observed among moderate-risk enrollees (those with chronic conditions such as diabetes) and low-risk enrollees (those without chronic or immunocompromising conditions).

**Conclusions:**

Controlling for individual characteristics attenuated the relationship between vulnerability and disease incidence overall and in most subgroups. Within MHSVI themes, ACP incidence is higher for the most versus least vulnerable counties based on Medical Vulnerability and Household Composition and Disability themes versus the overall MHSVI. Results for secondary outcomes (PP and IPD) follows similar patterns as for ACP but are weaker in magnitude and significance.

## Abstract (web summary)

Mohanty et al. examine the link between county-level Minority Health Social Vulnerability Index and rates of all-cause pneumonia, pneumococcal pneumonia, and invasive pneumococcal disease from 2016 to 2019 among publicly insured U.S. adults. Findings show that pneumococcal disease rates are higher in the most socially vulnerable counties.

## Plain Language Summary

Pneumonia is a common lung infection that can be especially serious for older adults and people with other health conditions. We used Medicare and Medicaid healthcare claims data from 2016 to 2019 to examine whether living in a more socially vulnerable county (based on information on income, disability, housing, and access to care for all people living in the county) was linked to higher pneumonia rates. We found that people living in more vulnerable areas had higher rates of pneumonia, especially if they were already at higher risk of disease due to health problems. These findings suggest that where people live can affect their chances of getting sick, even after accounting for individual health. Our results can help policymakers target resources and prevention efforts to the communities that need them most.

## Introduction

Pneumonia is a severe form of acute respiratory infection, with *Streptococcus pneumoniae*, a Gram-positive bacterium, a leading cause of bacterial pneumonia. In addition, *S. pneumoniae* is responsible for invasive pneumococcal disease (IPD) including meningitis and bacteremia and non-invasive disease such as non-bacteremic pneumococcal pneumonia (PP) and acute otitis media^1^. In the United States (U.S.), more than 1.5 million people are hospitalized annually due to community-acquired pneumonia^2^. Further, pneumococcal disease is associated with high clinical and economic burden^3^. However, pneumococcal disease incidence can vary greatly based on individual demographic and clinical factors^4^. For example, among U.S. adults, the risk of pneumococcal disease increases with age^5^ and with chronic and immunocompromising conditions^6–8^. The risk of pneumococcal disease is also higher among black adults—who also have higher age-adjusted mortality rates—due to higher prevalence of chronic and immunocompromising conditions than individuals of other races and lower vaccine uptake^9^.

Beyond individual factors, area-level social determinants of health (SDOH)—including non-medical factors such as housing, transportation, and healthcare access—can also play a role in disease burden. For example, during the COVID-19 pandemic, individuals in rural, remote, and low-income areas experienced more COVID-19 infections, in part because SDOH factors magnified longstanding disparities related to testing access, healthcare infrastructure, provider shortages, and risk messaging^10^. Despite the recognition that more than half of pneumonia-related hospitalizations may be due to community-acquired infections^11^, there is limited evidence on which community features affect pneumococcal disease burden.

The goal of our study was to characterize the relationship between area-level social vulnerability and all-cause pneumonia (ACP), PP, and IPD incidence across the U.S., using data for adults enrolled in Medicare and Medicaid. Medicare is a federal health insurance program with eligibility primarily based on age (65 or older), whereas eligibility for Medicaid is primarily based on low-income requirements and disability status. In 2019, these public health insurance programs covered 135 million people –72 million by Medicaid, 63 million by Medicare^12,13^.  We found that among high-risk Medicare and Medicaid enrollees, ACP incidence is higher in the most versus least socially vulnerable counties, whereas the opposite is observed among moderate-risk enrollees (those with chronic conditions such as diabetes) and low-risk enrollees (those without chronic or immunocompromising conditions), which can help state and local officials around the U.S. assess potential root causes of disparities in ACP, PP and IPD burden and identify strategies to better protect highly vulnerable individuals^14,15^.

## Methods

To describe the relationship between area-level social vulnerability and ACP, PP, and IPD burden among U.S. adults, we used a retrospective cohort design that combined individual-level data on ACP, PP, and IPD incidence from public insurance program claims with county-level data on social vulnerability from the Centers for Disease Control and Prevention (CDC). We analyzed Medicare data from January 1, 2016, to December 31, 2019, and Medicaid data from January 1, 2017, to December 31, 2019. Medicaid data from 2016 were excluded because data quality did not meet standards in several states. Furthermore, the measurement period ends in 2019 to exclude changes in disease incidence due to the COVID-19 pandemic. Medicare and Medicaid data were used under a data use agreement (DUA) between Mathematica and the Centers for Medicare & Medicaid Services (CMS). We requested data access from the Research Data Assistance Center (ResDAC). The use of CMS data under this DUA is under oversight at HealthMediaLab Institutional Review Board, which has deemed the research minimal risk and granted a waiver of informed consent.

We analyzed Medicare and Medicaid eligibility files and inpatient, outpatient, and prescription drug claims data maintained by CMS to (a) identify cohorts meeting our eligibility criteria, (b) define enrollee subgroups, and (c) calculate disease incidence. We used the Minority Health Social Vulnerability Index (MHSVI), a composite score released by the CDC^16^ to characterize county-level social vulnerability across all 50 U.S. states and the District of Columbia. The MHSVI we used was based on 2018 data and was made available in 2021. The index includes six themes: Socioeconomic Status, Household Composition and Disability, Minority Status and Language, Housing Type and Transportation, Health Care Infrastructure and Access, and Medical Vulnerability (Supplementary Table 1). The composite and theme indices are scored from 0 to 1, with higher values indicating higher social vulnerability.

By grouping individuals according to the vulnerability level of their county of residence, we assess whether living in a more versus less vulnerable area is associated with different disease incidence—even after accounting for individual demographic and clinical characteristics. The underlying premise is that area-level conditions can shape individuals’ health risks through structural and environmental pathways. For example, Housing Type and Transportation vulnerability may increase individuals’ exposure to infection through crowded living conditions or limit access to healthcare due to lack of transportation. Healthcare Infrastructure and Access reflects the density of local providers and facilities, which influences how easily individuals can obtain care. However, we do not hypothesize or test for specific directional effects as our analysis framework is interpretive, rather than causal.

### Study population

Our study population includes two cohorts: (1) adults ages 65+ years enrolled in Medicare fee-for-service or Medicare Advantage plans, and (2) adults ages 19–64 years enrolled in Medicaid. To be included in the analyses, Medicare enrollees had to be continuously enrolled for at least one year during the study period. Inclusion criteria for Medicaid enrollees required continuous enrollment for at least six months during the study period, residence in a state with reliable claims data, and residence in a county with available MHSVI data. We excluded the following states from the analysis: Florida, Maryland, New Hampshire, Rhode Island, Tennessee, and Vermont. We excluded states for which data elements critical for the analysis Data Quality Atlas classified as “high concern” or “unusable” in at least one year during the study period (Supplementary Table 2). We required a shorter continuous enrollment for Medicaid because Medicaid eligibility is determined by several criteria that change over time and vary by state, whereas with Medicare, most of the population includes individuals who are permanently eligible starting at age 65. These enrollment continuity and length restrictions were applied to avoid potential bias, whereby enrollees could have had fewer observable disease events because of enrollment gaps or a shorter length of enrollment. We excluded from the Medicaid cohort enrollees who were dually enrolled in Medicare and Medicaid because Medicare is the primary payer and we cannot capture disease episodes reliably in Medicaid alone. We also excluded Medicaid enrollees receiving long-term care services during the study period, since claims do not reliably capture procedure codes, which are used to classify risk of pneumococcal disease.

### Disease measures

Disease episodes were based on an ICD-10 diagnosis code for non-invasive ACP (due to any bacterial or viral cause), PP (caused by *S. pneumoniae*), or invasive pneumococcal disease (IPD), based on codes in Hu et al. 2022^17^ and Hu et al. 2023^18^ (Supplementary Data 5). We focused the primary analysis on ACP, which captures a broader pneumonia definition than PP. All-cause pneumonia better aligns with clinical practice, where pneumonia is diagnosed based upon clinical examination (particularly in the outpatient setting), without radiological confirmation or identification of a causative organism^19,20^. In secondary analyses, we also describe differences in results between ACP, PP, and IPD.

For each enrollee, the first claim with an ACP, PP, or IPD code triggered the start of a new episode for that disease. Given our focus on disease incidence, we excluded any disease episodes that occurred during the first month of enrollment, since claims for ACP, PP, or IPD in the first month could represent an ongoing disease episode. Further, multiple episodes of a given disease (such as ACP) that were less than 30 days apart, were considered a single disease episode, following the literature^17^.

We calculated disease incidence per 100,000 person-years (PY) by summing the number of distinct disease episodes across all enrollees and years, dividing the result by the sum of years each enrollee contributed during the study period minus the number of disease episodes, and multiplying by 100,000. For each cohort, we calculated disease incidence rates overall, by county, and within subgroups defined by age, race, ethnicity, and pneumococcal disease risk.

Enrollees with a high risk of pneumococcal disease were those with a code for cochlear implant, cerebrospinal fluid leak, immunocompromising diseases, asplenia, sickle cell disease, congenital or acquired asplenia, splenic dysfunction, chronic renal disease, cancer, human immunodeficiency virus (HIV), or organ transplant. Moderate-risk enrollees were those with a code for alcohol dependence, asthma, chronic heart disease, chronic liver disease, chronic lung disease, diabetes mellitus, or smoking. Those who did not meet either criterion were classified as low risk (Supplementary Data 6 and 7).

### Statistics and reproducibility

For each cohort, we first mapped county-level incidence rates and calculated the Moran’s I coefficient to descriptively explore spatial autocorrelation, that is, correlation in incidence rates between neighboring counties. The maps exclude counties with fewer than 120 person-years, to reduce noise.

To evaluate how incidence rates (for each outcome and demographic subgroup) varied by area-level social vulnerability (based on the overall MHSVI index and each of the six theme indices), we assigned counties an MHSVI quintile, which ranged from 1 as least vulnerable (0 ≤ MHSVI < 0.2) to 5 as most vulnerable (0.8 ≤ MHSVI ≤ 1). We then calculated disease incidence by MHSVI quintile by aggregating enrollee-level data for enrollees residing in counties assigned to each MHSVI quintile, based on enrollees’ most recent county of residence. We used a paired t-test to assess whether the incidence rate ratio (IRR), reflecting the ratio of disease incidence between counties in the fifth versus first MHSVI quintiles (Q5/Q1), differed significantly.

Lastly, we used Poisson regression to evaluate the relationship between area-level vulnerability and disease incidence, controlling for potential confounding by individual-level characteristics. We fit separate models for the Medicare and Medicaid cohorts. Our outcome, $E(y)$, is the count of disease episodes an enrollee experienced during the study period, with an offset term, $t$, that captures the number of person-years each enrollee contributed during the study period. Unadjusted models include only indicators for the MHSVI quintiles, with ${MHSVIQuintil}{e}_{x}$ representing a set of four binary variables indicating whether the beneficiary lives in a county in the second, third, fourth, or fifth (most vulnerable) quintile of the MHSVI. The omitted category is the first (least vulnerable) quintile. $\varepsilon$ is an idiosyncratic error term:

1 $$\log \left(\frac{E(y)}{t}\right)={\beta }_{0}+\,{\beta }_{1}* {MHSVI}{{Quintile}}_{\left\{2,3,4,5\right\}}+\varepsilon$$

The adjusted regressions include additional individual-level covariates to control for potential confounding, where $X$ is a vector of beneficiary covariates, including age group (for Medicaid: 19–49, 50–64 years; for Medicare: 65–74, 75–84, 85+ years), dual enrollment in Medicare and Medicaid (yes, no; controlled for in Medicare cohort models only), sex (male, female; Medicaid enrollees missing information on sex [n = 2000] were excluded from the models), pneumococcal disease risk status (low risk, moderate risk, high risk), race and ethnicity (non-Hispanic white, non-Hispanic black, Hispanic, Asian, Other, missing), and urbanicity (urban, suburban, rural):

2 $$\log \left(\frac{E(y)}{t}\right)=\,{\beta }_{0}+{\beta }_{1}* {MHSVI}{{Quintile}}_{\{2,3,4,5\}}+X\beta +\varepsilon$$

We defined urbanicity using the 2013 Rural-Urban Continuum Codes developed by the U.S. Department of Agriculture^21^, considering all metropolitan counties as urban, all non-metropolitan counties with 2500 people or more as suburban, and counties with fewer than 2500 people as rural (Supplementary Table 3).

In both the unadjusted and adjusted models, the key coefficients are ${\beta }_{0}$ and ${\beta }_{1}$. ${\beta }_{0}$ represents the log of the expected count of individuals with the disease among those in the reference category, that is, the least vulnerable quintile. ${\beta }_{1}$ represents the change in the log of the expected count of individuals with the disease among those who live in the most vulnerable quintile, relative to those who live in the least vulnerable quintile.

To assess the influence of potential confounders on the extent to which area-level vulnerability predicted disease incidence, we examined how the difference in disease incidence between MHSVI Q5 and MHSVI Q1 changed between the unadjusted and adjusted regression models. We also compared model fit before and after covariate adjustment using Akaike’s Information Criterion, for which smaller values represent a better model fit. Statistical significance was based on an unadjusted two-sided type I error rate of alpha = 0.05. All analyses were conducted using standard statistical software (SAS version 9.3 or later, and R version 4.4.0).

### Reporting summary

Further information on research design is available in the Nature Portfolio Reporting Summary linked to this article.

## Results

After applying eligibility criteria, a total of 56.9 million Medicare and 36.6 million Medicaid enrollees were included in the analysis (Supplementary Table 4). Below, we describe findings separately for the Medicare and Medicaid cohorts, focusing on ACP incidence. We also describe how results for PP and IPD incidence differed from results for ACP incidence.

### Medicare enrollees

#### Cohort characteristics and all-cause pneumonia incidence

Of the 56.9 million Medicare enrollees analyzed, most were female (55%), non-Hispanic white (81%), 65 to 74 years of age (51%), at high risk for pneumococcal disease (51%), and lived in an urban area (83%) (Table 1). All-cause pneumonia incidence was 7910 per 100,000 PY overall and varied by demographic and clinical features. Higher incidence rates than the overall rate among enrollees were observed in the following groups: males (8119 per 100,000 PY); non-Hispanic white participants (8143 per 100,000 PY), those aged 85+ (16,003 per 100,000 PY), those at high risk for pneumococcal disease (11,571 per 100,000 PY), and residents of a rural or suburban area (8588 and 8520 per 100,000 PY, respectively) (Table 1). Black and Hispanic enrollees had lower ACP incidence than white enrollees, regardless of age, in the low- and moderate-risk groups, but higher ACP incidence in the high-risk group (Supplementary Table 5).

**Table 1:** Characteristics of Medicare enrollees (2016–2019)

Group	Number of enrollees in group	Percentage in group	ACP incidence rate (per 100,000 PY)	PP incidence rate (per 100,000 PY)	IPD incidence rate (per 100,000 PY)
Overall	56,909,851	100.0%	7910	42.0	41.3
Sex					
Male	25,602,064	45.0%	8119	43.2	45.0
Female	31,307,787	55.0%	7742	41.1	38.3
Race and ethnicity					
White, non-Hispanic	46,302,403	81.4%	8143	43.0	40.9
Black, non-Hispanic	5,223,475	9.2%	7680	36.6	51.3
Hispanic	1,400,384	2.5%	7320	45.2	42.4
Asian	1,485,490	2.6%	6452	45.9	33.8
Other	1,353,925	2.4%	6576	40.3	42.0
Missing	1,144,174	2.0%	3597	19.5	19.2
Age					
65–74	28,856,668	50.7%	4757	26.9	29.5
75–84	17,948,838	31.5%	8605	46.0	44.4
85+	10,104,112	17.8%	16,003	79.2	70.8
Risk of pneumococcal disease					
Low risk	9,859,367	17.3%	416	1.9	1.1
Moderate risk	17,945,773	31.5%	5635	31.8	26.6
High risk	29,104,711	51.1%	11,571	60.5	62.4
Urbanicity					
Urban	46,998,726	82.6%	7780	40.6	40.3
Suburban	8,782,849	15.4%	8520	48.7	45.9
Rural	1,128,276	2.0%	8588	48.2	45.9

Rows for gender and age do not sum to overall number of enrollees because those with missing age (*N* = 264) and gender (*N* = 6) were excluded from the table. The analysis included enrollees in Medicare fee-for-service and Medicare Advantage plans.

*ACP* All-cause pneumonia, *PP* pneumococcal pneumonia, *IPD* invasive pneumococcal disease, *PY* person-years.

**Fig. 1:**
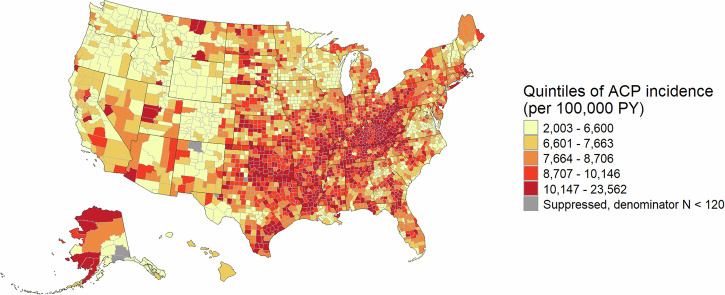
Unadjusted county-level ACP incidence among Medicare enrollees (2016–2019). The choropleth map shows U.S. counties color-coded based on ACP incidence rate quintile. We suppressed data for six counties with fewer than 120 person-years. Yellow counties: 2003–6600 per 100,000 PY; light orange counties: 6601–7663; dark orange: 7664–8706; bright red: 8707–10,146; dark red: 10,147–23,562; gray: suppressed due to denominator *N* < 120. *ACP* All-cause pneumonia, *PY* person-years.

County-level ACP incidence ranged from 2003 to 23,562 per 100,000 PY (Fig. 1). ACP incidence was highest in the Appalachian region and the central South, and lowest in the Northwest. There was significant spatial clustering in ACP incidence rates (Supplementary Table 6), indicating that neighboring counties had more similar disease rates than more distant counties.

#### All-cause pneumonia incidence by social vulnerability

When counties were grouped into quintiles by their level of social vulnerability, we observed higher ACP incidence as the MHSVI quintile increased (Supplementary Table 7). Overall, enrollees in the most socially vulnerable counties had a 16% higher ACP incidence rate than those in the least socially vulnerable counties (IRR = 1.16, *p* < 0.0001). This pattern was observed in all age groups, most race and ethnicity groups, and in high-risk enrollees (Table 2). Enrollees at high risk for pneumococcal disease had both a higher ACP incidence and a greater difference in incidence between those living in the most versus least socially vulnerable counties, compared to the overall cohort (IRR = 1.23, *p* < 0.0001). However, we found that the relationship between social vulnerability and ACP incidence was reversed—there was a lower ACP incidence in the most compared to least socially vulnerable counties—among Medicare enrollees who were low risk (IRR = 0.69) or moderate risk (IRR = 0.96) for pneumococcal disease. We also noted that, although Hispanic and Asian enrollees had a lower ACP incidence than the overall cohort, the disparity in incidence between the most versus least socially vulnerable counties was larger among both of these subgroups than in the overall cohort (IRR = 1.36 and 1.37 for Asian and Hispanic enrollees, respectively).

**Table 2:** Unadjusted difference in ACP incidence among Medicare enrollees, by social vulnerability (2016–2019)

Group	ACP incidence (per 100,000 PY): MHSVI Q1 counties	ACP incidence (per 100,000 PY): MHSVI Q5 counties	Unadjusted IRR (Q5 / Q1)	95% Confidence Interval around IRR	IRR *p*-value
Overall	7073	8203	1.16***	(1.157, 1.162)	<0.0001
Age					
65–74	4074	5104	1.25***	(1.247, 1.258)	<0.0001
75–84	7739	8973	1.16***	(1.155, 1.164)	<0.0001
85+	14,675	16,252	1.11***	(1.103, 1.112)	<0.0001
Race and ethnicity					
White, non-Hispanic	7179	8489	1.18***	(1.180, 1.185)	<0.0001
Black, non-Hispanic	6967	7867	1.13***	(1.104, 1.155)	<0.0001
Hispanic	5618	7683	1.37***	(1.287, 1.453)	<0.0001
Asian	5163	7020	1.36***	(1.303, 1.419)	<0.0001
Other	7163	7121	0.99	(0.970, 1.019)	0.643
Risk of pneumococcal disease					
Low risk	518	357	0.69***	(0.674, 0.705)	<0.0001
Moderate risk	5638	5426	0.96***	(0.958, 0.967)	<0.0001
High risk	10,079	12,436	1.23***	(1.231, 1.237)	<0.0001

Notes: The analysis included enrollees in Medicare fee-for-service and Medicare Advantage plans. Ns presented in Table 1.

* *p*-value < 0.05; ** *p*-value < 0.01; *** *p*-value < 0.001

*ACP* All-cause pneumonia, *IRR* incidence rate ratio, *MHSVI* Minority Health Social Vulnerability Index, *PY* person-years, *Q1* least vulnerable quintile, *Q5* most vulnerable quintile.

After controlling for individual enrollee characteristics, we found that the overall relationship between social vulnerability and ACP incidence was attenuated, but remained statistically significant (adjusted IRR = 1.12 versus 1.16 unadjusted) (Tables 2 and 3). Further, we found that regression adjustment reversed the direction of the relationship for moderate-risk enrollees, such that ACP incidence was slightly higher in the most versus least vulnerable counties after adjustment (IRR = 1.04, *p* < 0.0001), but slightly lower before adjustment (IRR = 0.96, *p* < 0.0001). Among low-risk enrollees, the relationship remained relatively unchanged (adjusted IRR = 0.74 versus 0.69 unadjusted) (Tables 2 and 3). In models based on the full Medicare sample and in each subgroup analysis, adjusting for individual enrollee characteristics improved model fit (Supplementary Table 8).

**Table 3:** Adjusted differences in ACP incidence among Medicare enrollees, by social vulnerability (2016–2019)

Group	Number of enrollees included in model	Regression-adjusted IRR (Q5/Q1)	95% Confidence Interval around IRR	IRR *p*-value
Overall	56,909,618	1.12***	(1.12, 1.12)	<0.0001
Age				
65–74	28,856,668	1.13***	(1.12, 1.13)	<0.0001
75–84	17,948,838	1.12***	(1.12, 1.13)	<0.0001
85+	10,104,112	1.10***	(1.10, 1.11)	<0.0001
Race and ethnicity				
White, non-Hispanic	46,302,284	1.12***	(1.11, 1.12)	<0.0001
Black, non-Hispanic	5,223,404	1.06***	(1.03, 1.08)	<0.0001
Hispanic	1,400,368	1.12***	(1.05, 1.19)	0.0004
Asian	1,485,480	1.13***	(1.08, 1.18)	<0.0001
Other	1,353,908	1.12***	(1.09, 1.15)	<0.0001
Risk of pneumococcal disease				
Low risk	9,859,319	0.74***	(0.72, 0.76)	<0.0001
Moderate risk	17,945,748	1.04***	(1.03, 1.04)	<0.0001
High risk	29,104,551	1.16***	(1.15, 1.16)	<0.0001

The analysis included enrollees in Medicare fee-for-service and Medicare Advantage plans. For enrollees with missing race or ethnicity, we included a missing value indicator in the model, but did not separately estimate differences in ACP incidence for that group.

* *p*-value < 0.05, ** *p*-value < 0.01, *** *p*-value < 0.001.

*ACP* All-cause pneumonia, *IRR* incidence rate ratio, *MHSVI* Minority Health Social Vulnerability Index, *PY* person-years, *Q1* least vulnerable quintile, *Q5* most vulnerable quintile.

**Fig. 2:**
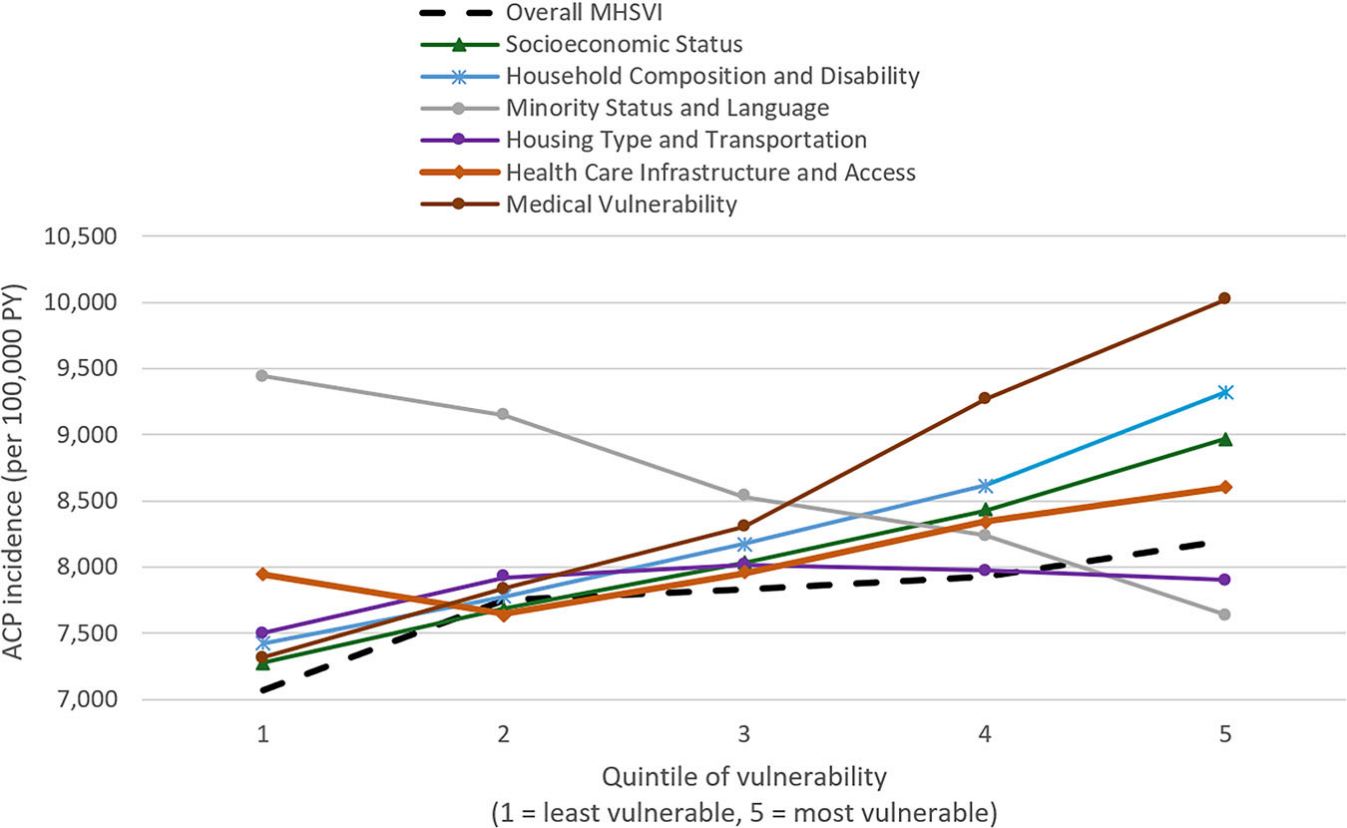
Unadjusted ACP incidence among Medicare enrollees, by overall MHSVI and theme quintile (2016–2019). The graph plots the ACP incidence among counties in each MHSVI quintile, with one line per MHSVI index (the overall index and six theme indices). Analyses included enrollees in Medicare fee-for-service and Medicare Advantage plans. See Supplementary Data 2 for the underlying data. Dashed line: Overall MHSVI; green line: socioeconomic status; blue line: household composition and disability; gray line: minority status and language; purple line: housing type and transportation; orange line: health care infrastructure and access; brown line: medical vulnerability. ACP All-cause pneumonia, MHSVI Minority Health Social Vulnerability Index, PY person-years.

When we examined how the relationship between social vulnerability and ACP incidence varied across MHSVI themes, we observed higher ACP incidence in higher MHSVI quintiles for five out of six subthemes—all but the Minority Status and Language theme (Fig. 2). The largest disparities in ACP incidence were observed for the Medical Vulnerability theme index (IRR = 1.37, *p* < 0.0001) and the Household Composition and Disability theme index (IRR = 1.25, *p* < 0.0001); ACP incidence varied least by the Housing Type and Transportation vulnerability theme index. When vulnerability was based on the Minority Status and Language theme, the pattern was reversed, whereby counties that were more racially or ethnically vulnerable had a lower ACP incidence than those who were less vulnerable on this dimension (IRR = 0.81, *p* < 0.0001) (Supplementary Table 9).

#### Pneumococcal pneumonia incidence

Pneumococcal pneumonia incidence was 42 per 100,000 PY overall and varied by age, race and ethnicity, and disease risk (Supplementary Table 10), as well as by combinations of those characteristics (Supplementary Table 11). Geographic patterns in county-level PP incidence generally resembled patterns observed with ACP (Supplementary Fig. 1a).

#### Pneumococcal pneumonia incidence by social vulnerability

Relationships between social vulnerability and PP incidence also followed similar patterns as observed for ACP incidence, though were weaker in magnitude and significance (Supplementary Table 10). Overall, Medicare enrollees residing in the most vulnerable counties had a 10% higher unadjusted PP incidence rate (IRR = 1.10, *p* < 0.0001) than those in the least vulnerable counties (Supplementary Table 12). Adjustment for individual-level enrollee demographic and clinical characteristics reduced the level of disparity in the MHSVI overall index in the full sample (adjusted IRR = 1.09, *p* < 0.0001) and within several subgroups. Although ACP incidence was higher among Hispanic participants residing in the most versus least vulnerable counties (adjusted IRR = 1.12, *p* < 0.0001), PP incidence was lower (unadjusted IRR = 0.33, *p* < 0.0001), even after adjustment for individual enrollee characteristics (adjusted IRR = 0.27, *p* < 0.0001). The magnitude of disparity was greatest when vulnerability was based on indices for the Medical Vulnerability theme (IRR = 1.35, *p* < 0.0001), Household Composition and Disability theme (IRR = 1.42, *p* < 0.0001), and Socioeconomic Status theme (IRR = 1.40, *p* < 0.0001) (Supplementary Table 13; Supplementary Fig. 2a). As with ACP, the direction of the relationship was reversed (with a lower PP incidence among more vulnerable counties) when vulnerability was based on the Minority Status and Language theme index (IRR = 0.78, *p* < 0.0001).

#### Invasive pneumococcal disease incidence

Invasive pneumococcal disease incidence was 41 per 100,000 PY overall and varied by age, race and ethnicity, and risk status (Supplementary Table 14), as well as by combinations of those characteristics (Supplementary Table 15). Geographic patterns in county-level IPD incidence generally resembled patterns observed with ACP and PP (Supplementary Fig. 3a). Relationships between the overall MHSVI index and IPD incidence followed a somewhat similar pattern as observed for ACP incidence, but estimates were much smaller in magnitude and weaker in significance (Supplementary Table 15). Overall, Medicare enrollees in the most vulnerable counties had a 11% higher unadjusted IPD incidence rate (IRR = 1.11, *p* < 0.0001) than those in the least vulnerable counties (Supplementary Table 16). However, adjustment for individual-level enrollee demographic and clinical characteristics eliminated this disparity in IPD incidence in the full cohort (adjusted IRR = 1.00, *p* = 0.95) and among all subgroups analyzed. No clear trend emerged when we examined the relationship between IPD incidence and vulnerability based on quintiles of the MHSVI theme indices (Supplementary Table 17; Supplementary Fig. 4a).

### Medicaid enrollees

#### Cohort characteristics and all-cause pneumonia incidence

Of the 36.6 million Medicaid enrollees analyzed, most were female (58%), 19 to 49 years of age (80%), at low risk for pneumococcal disease (77%), and lived in an urban area (86%) (Table 4). More Medicaid than Medicare enrollees were part of a racial or ethnic minority group (24% versus 14% were non-white and non-Hispanic, and 20% versus 2.5% were Hispanic, respectively). All-cause pneumonia incidence was 1819 per 100,000 PY overall and varied by demographic and clinical features: ACP incidence rates were higher than the overall rate among enrollees who were male (1865 per 100,000 PY); non-Hispanic white or Other race (2273 and 2189 per 100,000 PY, respectively), aged 50 to 64 (3597 per 100,000 PY), at high risk or moderate risk for pneumococcal disease (5137 and 4645 per 100,000 PY, respectively); and lived in a rural or suburban area (2430 and 2312 per 100,000 PY, respectively). Non-Hispanic black enrollees had lower ACP incidence than non-Hispanic white enrollees, regardless of age, in the low- and moderate-risk groups, but higher ACP incidence in the high-risk group (Supplementary Table 5).

**Table 4:** Characteristics of Medicaid enrollees (2017–2019)

Group	Number of enrollees in group	Percentage in group	ACP incidence rate (per 100,000 PY)	PP incidence rate (per 100,000 PY)	IPD incidence rate (per 100,000 PY)
Overall	36,579,701	100.0%	1819	9.6	14.1
Sex					
Male	15,257,731	41.7%	1865	10.6	18.2
Female	21,319,969	58.3%	1788	8.9	11.4
Race and ethnicity					
White, non-Hispanic	13,417,380	36.7%	2273	12.6	16.7
Black, non-Hispanic	5,988,401	16.4%	1916	10.0	16.9
Hispanic	7,373,733	20.2%	1188	5.4	8.4
Asian	1,990,600	5.4%	918	4.2	4.3
Other	824,048	2.3%	2189	12.4	31.6
Missing	6,985,539	19.1%	1748	9.2	13.6
Age					
19–49	29,383,244	80.3%	1362	5.8	8.6
50–64	7,196,457	19.7%	3597	24.3	35.5
Risk of pneumococcal disease					
Low risk	27,973,467	76.5%	848	3.6	5.8
Moderate risk	7,330,617	20.0%	4645	27.0	36
High risk	1,275,617	3.5%	5137	30.5	56.5
Urbanicity					
Urban	31,578,103	86.3%	1736	9.0	13.7
Suburban	4,512,961	12.3%	2312	12.9	16.8
Rural	488,637	1.3%	2430	13.9	16.4

The table shows disease incidence per 100,000 person-years. Rows for gender do not sum to overall number of enrollees, because those with missing information (*N* = 2001) were excluded from the table.

*ACP* All-cause pneumonia; *PP* pneumococcal pneumonia, *IPD* invasive pneumococcal disease, *PY* person-years.

**Fig. 3:**
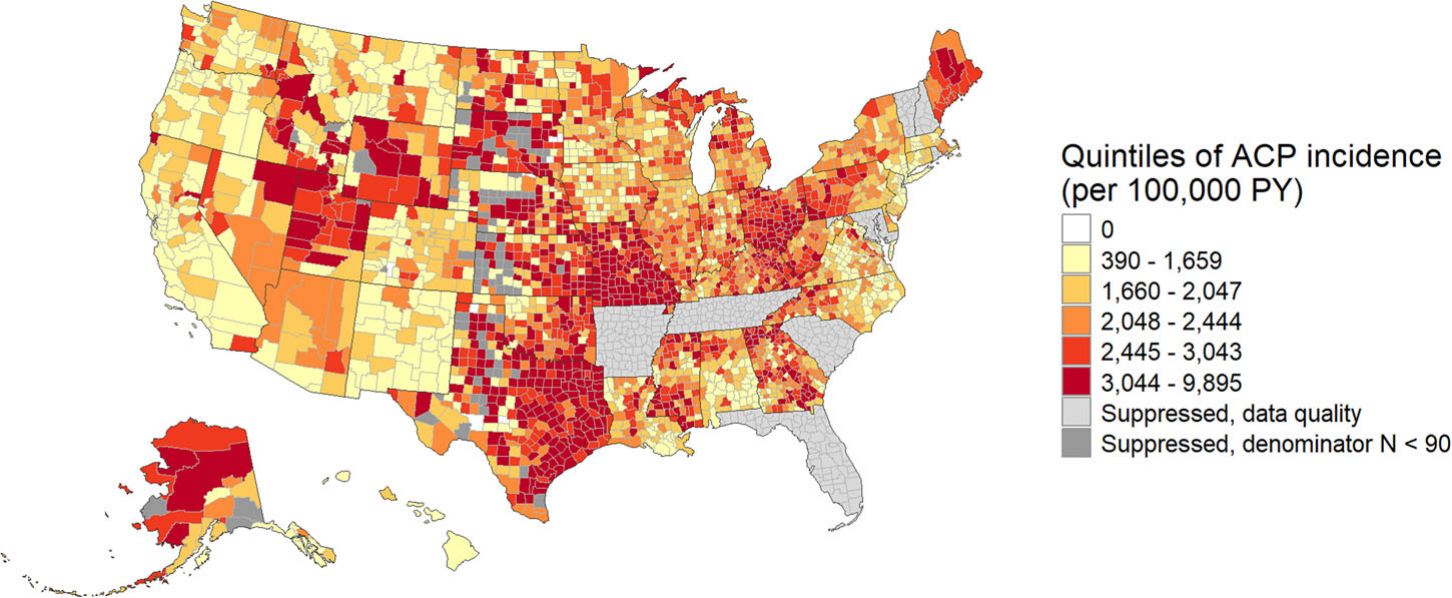
Unadjusted county-level ACP disease incidence among Medicaid enrollees (2017–2019). The choropleth map shows U.S. counties color-coded based on ACP incidence rate quintile. Yellow counties: 390–1659 per 100,000 PY; light orange counties: 1660–2047; dark orange: 2048–2444; bright red: 2445–3043; dark red: 3044-9895; light gray: suppressed due to data quality; dark gray: suppressed due to denominator *N* < 90. Data from Arkansas, Florida, Maryland, New Hampshire, Rhode Island, South Carolina, Tennessee, and Vermont were suppressed due to data quality below desired standards. We also suppressed data for 84 counties with fewer than 90 person-years. Very small and very large disease incidence rates are plausible for counties with small populations. ACP All-cause pneumonia, PY person-years.

County-level ACP incidence ranged from 0 to 9895 per 100,000 PY (Fig. 3). There was significant spatial clustering in ACP incidence rates among the states included in analysis (Supplementary Table 6), with higher rates throughout the Great Plains and extending through Texas, and lower rates on both coasts (Fig. 3).

#### All-cause pneumonia incidence by social vulnerability

When counties were grouped by their social vulnerability, we saw lower ACP incidence as the MHSVI quintile increased (Supplementary Table 7). Overall, enrollees in the most socially vulnerable counties had a lower ACP incidence rate than those in the least socially vulnerable counties (IRR = 0.79, *p* < 0.0001). This pattern was upheld across most subgroups, with some exceptions: the relationship was reversed—with a higher ACP incidence rate among those in the most versus least socially vulnerable counties—among non-Hispanic black (IRR = 1.09) and high-risk (IRR = 1.29) Medicaid enrollees (Table 5).

**Table 5:** Unadjusted difference in ACP incidence among Medicaid enrollees, by social vulnerability (2017–2019)

Group	ACP incidence (per 100,000 PY): MHSVI Q1 counties	ACP incidence (per 100,000 PY): MHSVI Q5 counties	Unadjusted IRR (Q5 / Q1)	95% Confidence Interval around IRR	IRR *p*-value
Overall	2095	1650	0.79***	(0.78, 0.80)	<0.0001
Age					
19–49	1653	1204	0.73***	(0.72, 0.74)	<0.0001
50–64	3807	3377	0.89***	(0.87, 0.90)	<0.0001
Race and ethnicity					
White, non-Hispanic	2192	2145	0.98***	(0.97, 0.99)	0.0004
Black, non-Hispanic	1742	1902	1.09**	(1.03, 1.15)	0.0015
Hispanic	1704	1132	0.66***	(0.64, 0.69)	<0.0001
Asian	1097	934	0.85**	(0.77, 0.95)	0.0029
Other	2333	2039	0.87***	(0.82, 0.93)	<0.0001
Risk of pneumococcal disease					
Low risk	1049	755	0.72***	(0.71, 0.73)	<0.0001
Moderate risk	4840	4558	0.94***	(0.93, 0.95)	<0.0001
High risk	4277	5510	1.29***	(1.25, 1.33)	<0.0001

The table shows disease incidence per 100,000 person-years. Ns presented in Table 4.

*p-value < 0.05; **p-value < 0.01; ***p-value < 0.001.

*ACP* All-cause pneumonia, *IRR* incidence rate ratio, *MHSVI* Minority Health Social Vulnerability Index, *PY* person-years, *Q1* least vulnerable quintile; *Q5* most vulnerable quintile.

Controlling for individual enrollee characteristics changed the relationship between social vulnerability and ACP incidence in some groups (Tables 5 and 6). Overall, the IRR changed from 0.79 to 1.02 after adjustment. The relationship was also reversed among enrollees who were 50 to 64 years of age (adjusted IRR = 1.11 versus 0.89 unadjusted), moderate risk for pneumococcal disease (IRR = 1.06 versus 0.94 unadjusted), and non-Hispanic white (adjusted IRR = 1.04 versus 0.98 unadjusted). Regression adjustment reduced the relationship between social vulnerability and ACP incidence among enrollees who were 19 to 49 years of age (adjusted IRR = 0.97 versus 0.73 unadjusted), Hispanic (adjusted IRR = 0.90 versus 0.66 unadjusted) and low-risk enrollees (adjusted IRR = 0.91 versus 0.72 unadjusted) but had little impact on the relationship among non-Hispanic black, Asian, and high-risk enrollees. All findings in Tables 5 and 6 were statistically significant, except for non-Hispanic black enrollees and enrollees of Other race in adjusted analyses. Overall and in subgroup analyses, adjusting for individual enrollee characteristics improved model fit (Supplementary Table 8).

**Table 6:** Adjusted differences in ACP incidence among Medicaid enrollees, by social vulnerability (2017–2019)

Group	Number of enrollees included in model	Regression-adjusted IRR (Q5/Q1)	95% Confidence Interval around IRR	IRR *p*-value
Overall	36,577,700	1.02***	(1.01, 1.03)	0.0002
Age				
19–49	29,381,308	0.97***	(0.96, 0.98)	<0.0001
50–64	7,196,392	1.11***	(1.09, 1.12)	<0.0001
Race and ethnicity				
White, non-Hispanic	13,417,270	1.04***	(1.03, 1.06)	<0.0001
Black, non-Hispanic	5,988,379	1.03	(0.97, 1.08)	0.34
Hispanic	7,373,713	0.90***	(0.86, 0.93)	<0.0001
Asian	1,990,595	0.89*	(0.80, 0.99)	0.044
Other	824,044	1.00	(0.94, 1.07)	0.95
Risk of pneumococcal disease				
Low risk	27,971,534	0.91***	(0.89, 0.92)	<0.0001
Moderate risk	7,330,565	1.06***	(1.05, 1.07)	<0.0001
High risk	1,275,601	1.24***	(1.20, 1.28)	<0.0001

The analysis included enrollees in Medicare fee-for-service and Medicare Advantage plans.

*p-value < 0.05; ** *p*-value < 0.01; *** *p*-value < 0.001.

*ACP* All-cause pneumonia, *IRR* incidence rate ratio, *MHSVI* Minority Health Social Vulnerability Index, *PY* person-years, *Q1* least vulnerable quintile, *Q5* most vulnerable quintile.

**Fig. 4:**
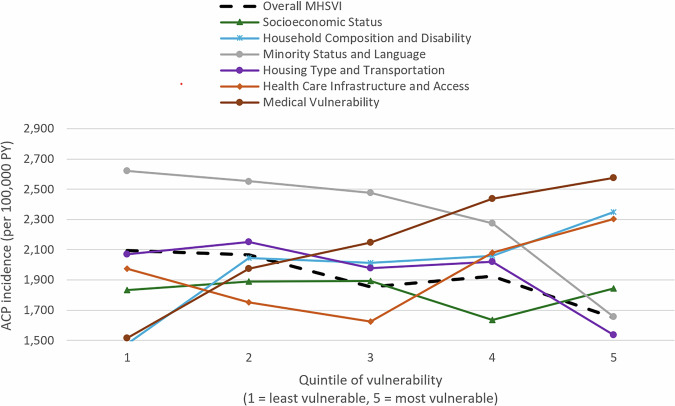
Unadjusted ACP incidence by MHSVI overall and theme quintiles among Medicaid enrollees (2017–2019). The graph plots the ACP incidence among counties in each MHSVI quintile, with one line per MHSVI index (the overall index and six theme indices). Analyses included Medicaid enrollees. See Supplementary Data 4 for the underlying data. Dashed line: Overall MHSVI; green line: socioeconomic status; blue line: household composition and disability; gray line: minority status and language; purple line: housing type and transportation; orange line: health care infrastructure and access; brown line: medical vulnerability. ACP All-cause pneumonia, MHSVI Minority Health Social Vulnerability Index, PY person-years.

The relationship between social vulnerability and ACP incidence varied greatly by vulnerability type, based on MHSVI theme indices (Fig. 4; Supplementary Table 9). ACP incidence was lower for the most versus least vulnerable counties when vulnerability was based on the Minority Status and Language (IRR = 0.63, *p* < 0.0001) and Housing Type and Transportation (IRR = 0.74, *p* < 0.0001) theme indices, but higher when vulnerability was based on the Medical Vulnerability (IRR = 1.70, *p* < 0.0001) and Household Composition and Disability theme indices (IRR = 1.59, *p* < 0.0001). We found little difference in ACP incidence among counties with a high versus low Socioeconomic Status vulnerability (IRR = 1.01, *p* = 0.17), likely because Medicaid enrollees are, by definition, a low-income group.

#### Pneumococcal pneumonia incidence

Pneumococcal pneumonia incidence was 9.6 per 100,000 PY overall and varied by age, race and ethnicity, and pneumococcal disease risk status (Supplementary Table 10) as well as by combinations of those characteristics (Supplementary Table 11). County-level PP incidence was low (often 0) in Midwestern counties (Supplementary Fig. 1b).

#### Pneumococcal pneumonia incidence by social vulnerability

Relationships between social vulnerability and PP incidence generally followed similar patterns as observed for ACP incidence, though were weaker in magnitude and significance. Overall, Medicaid enrollees residing in the most socially vulnerable counties had a lower PP incidence rate (IRR = 0.82, *p* = 0.0041) than those in the least socially vulnerable counties (Supplementary Table 12). Adjustment for individual-level enrollee demographic and clinical characteristics had varying effects. Overall, regression adjustment changed the direction and eliminated the significance of the relationship between PP incidence and social vulnerability (adjusted IRR = 1.11, *p* = 0.14, versus unadjusted IRR = 0.82) (Supplementary Table 12). However, among enrollees 50 to 64 years of age, regression adjustment increased the strength and significance of the relationship between PP incidence and social vulnerability (IRR = 1.35, *p* = 0.0058, versus unadjusted IRR = 0.99). The magnitude of difference was greatest when vulnerability was based on the Minority Status and Language theme index (IRR = 0.61, *p* < 0.0001) and the Housing Type and Transportation theme index (IRR = 0.76, *p* = 0.015). However, the direction of the relationship was reversed (with a higher PP incidence among the most vulnerable counties) when vulnerability was based on the other four MHSVI theme indices (Supplementary Table 13; Supplementary Fig. 2b).

#### Invasive pneumococcal disease incidence

Invasive pneumococcal disease incidence was 14.1 per 100,000 PY overall and varied by age, race, and ethnicity, and pneumococcal disease risk status (Supplementary Table 14), as well as by combinations of those characteristics (Supplementary Table 15). As with PP, county-level IPD incidence was low, and often 0, in many Midwestern counties (Supplementary Fig. 3b).

#### Invasive pneumococcal disease incidence by social vulnerability

Relationships between social vulnerability (based on the overall MHSVI index) and the unadjusted IPD incidence were not statistically significant overall (IRR = 0.91, *p* = 0.095) nor in most subgroups analyzed (Supplementary Table 16). The one exception was that IPD incidence was significantly lower among Asian living in the most versus least vulnerable counties (IRR = 0.26, *p* = 0.033), and this difference persisted even after adjusting for individual-level enrollee demographic and clinical characteristics (Supplementary Table 16). Disparities in IPD incidence became statistically significant when vulnerability was based on any of the MHSVI theme indices other than the Socioeconomic Status theme index (Supplementary Table 17; Supplementary Fig. 4b). Invasive pneumococcal disease incidence was also lower for those living in the most versus least vulnerable counties when vulnerability was based on the Minority Status and Language theme index (IRR = 0.83, *p* = 0.005) and the Housing Type and Transportation theme index (IRR = 0.83, *p* = 0.005). The direction of the relationship was reversed (with a higher IPD incidence in the most vulnerable counties) when vulnerability was based on Household Composition and Disability, Health Care Infrastructure and Access, or Medical Vulnerability.

## Discussion

Our study yields comprehensive estimates of disparities in ACP, PP, and IPD incidence by area-level social vulnerability among enrollees in U.S. public health insurance programs. Throughout our analysis, we report both unadjusted and adjusted incidence rate ratios to provide complementary insights. Unadjusted comparisons reflect the total observed differences in disease burden between individuals living in more versus less vulnerable counties, inclusive of both individual- and area-level factors. Adjusted estimates, on the other hand, isolate the association between county-level vulnerability and individual disease incidence, controlling for demographics, pneumococcal disease risk status, and urbanicity. We interpret the changes between these models as informative of whether observed disparities are driven primarily by individual characteristics or by contextual differences in community vulnerability.

All-cause pneumonia, PP and IPD incidence rates were higher among Medicare than Medicaid enrollees and varied significantly between counties with the highest and lowest levels of social vulnerability. The specific measure of vulnerability used strongly influenced results. Disparities in ACP disease incidence based on the overall MHSVI index were heightened when social vulnerability was based on the Medical Vulnerability or Household Composition and Disability theme indices instead. Thus, the strong association between overall MHSVI and pneumonia incidence was partly driven by the Medical Vulnerability theme, which reflects county-level prevalence of chronic conditions—such as diabetes, chronic respiratory disease, and cardiovascular disease—that are also direct risk factors for pneumonia. While this overlap may contribute to the observed association, we note that the Medical Vulnerability index is derived from the full county population, whereas our analysis is restricted to Medicare or Medicaid enrollees. As such, the theme reflects contextual disease burden rather than individual-level risk among the study population. Still, we recognize that including Medical Vulnerability in the composite index may complicate interpretation, particularly when distinguishing the role of clinical risk clustering from broader structural or environmental disadvantage. We also found some notable differences between the two cohorts. Among Medicare enrollees, ACP incidence was higher in the most versus least vulnerable counties (IRR > 1.0) in most subgroups analyzed. By contrast, among Medicaid enrollees, ACP incidence was lower in the most versus least vulnerable counties among enrollees who were aged 19 to 49, Hispanic, or Asian.

It is challenging to compare disease incidence for Medicare and Medicaid cohorts for several reasons, including: the characteristics of the two populations differ considerably, Medicaid enrollees lose and regain eligibility, Medicaid analysis excludes several states, Medicare data are of better quality, and the measurement period covers slightly different periods (2016 is included in Medicare, but not in Medicaid analyses). The main reason for differences in disease incidence between the two cohorts are differences in pneumococcal disease risk, since Medicare enrollees are older than Medicaid enrollees and more likely to be at high risk for pneumococcal disease (51% versus 3.5%, respectively). Among high-risk and moderate-risk Medicare and Medicaid enrollees, we saw a higher adjusted ACP incidence among the most versus least socially vulnerable counties. Likewise, among low-risk Medicare and Medicaid enrollees, we observed the opposite relationship, with a lower adjusted ACP incidence among the most versus least socially vulnerable counties. We also found that the strength of the relationship between social vulnerability and ACP incidence was similar between Medicare enrollees (adjusted IRR = 1.12 overall) and Medicaid enrollees aged 50 to 64 (adjusted IRR = 1.11), for whom the CDC’s Advisory Committee on Immunization Practices recommends vaccination^22^.

Our observation of a lower ACP disease incidence in more vulnerable counties among low-risk Medicare enrollees and among Medicaid enrollees who were low-risk, ages 19 to 49, Hispanic, or Asian may be counterintuitive. One potential explanation is that vulnerability may directly impact individuals’ access to healthcare and care-seeking behavior, especially for less severe illnesses like mild ACP or PP, due to lack of healthcare infrastructure, lack of transportation, or other factors^23,24^. This can result in underdiagnosis and fewer encounters captured in claims data, particularly among lower-risk individuals who may not engage with the healthcare system regularly. In contrast, high-risk enrollees—who are more likely to receive routine care—may be more consistently diagnosed. The variable relationship we observed between ACP incidence and quintiles of MHSVI theme indices suggest a potentially complex set of interactions between different root-causes of vulnerability and disease.

Our findings align with the literature in some ways. Consistent with the large body of research on how social factors influence health^25^, we found a strong, direct relationship between higher levels of area-level social vulnerability and higher pneumococcal disease incidence. However, we noted that the relationship was reversed when vulnerability was based on the Minority Status and Language theme, with lower ACP, PP, and IPD incidence among counties that were more socially vulnerable. The inverted relationship when vulnerability is based on the Minority Status and Language theme aligns with research by Saelee et al. (2023)^26^. They found that counties with higher vulnerability generally had lower COVID-19 vaccination coverage, but that counties with greater MHSVI Minority Status and Language vulnerability had *greater* COVID-19 vaccination coverage. One explanation Saelee et al. (2023)^26^ offered is that efforts by the CDC, local and state health departments may have reduced vaccine hesitancy and improved access to vaccination in racial and ethnic minority communities. We saw a higher IPD incidence among males compared to females, consistent with findings from de St. Maurice (2016)^27^. We also saw that IPD incidence was highest among non-Hispanic black enrollees^28^, and higher among Medicare enrollees residing in counties with higher Socioeconomic Status vulnerability, consistent with findings from Chen et al. (1998)^29^.

The fact that the relationship between social vulnerability and disease incidence was stronger before adjusting for individual demographic and clinical factors suggests that more vulnerable individuals tend to live in areas classified as vulnerable. Further, it suggests that observed differences in disease incidence between more and less vulnerable counties are driven, at least in part, by differences in the characteristics of their residents, rather than by place-specific social, infrastructural, or environmental factors. However, the relationship with area-level vulnerability remained even after regression adjustment in some subgroups (such as those with high risk for pneumococcal disease), suggesting that area-level factors could magnify the impact of individual factors on pneumococcal disease risk.

Our study had a few limitations. Our results may not generalize to the broader Medicaid population because of the restrictions we applied related to continuous enrollment, dual enrollment, and long-term care receipt, and the restriction to states with high-quality data. Our continuous enrollment requirement reduced the level of “churn” (when enrollees disenroll and re-enroll within a short period of time), which is common among Medicaid enrollees, and may have skewed the population towards individuals with more stable income and access to healthcare^30^, excluding working individuals whose monthly incomes fluctuate. Further, data quality issues may have impacted our analyses. Specifically, the varying quality of Medicare managed care claims data (by plan and over time, with some plans missing all encounter data), and Medicaid data quality issues that persisted even after excluding states with poor quality data may have introduced measurement error in disease incidence across geographic areas. In addition, excluding states due to insufficient data quality may have reduced the representation of certain vulnerable populations in our analysis, which could limit the generalizability of findings and hinder identification of disparities in those areas. Comparisons of disease incidence at the county level based on the maps generated should be made with caution, since some counties have small population counts. Also, race and ethnicity subgroup results for the Medicaid cohort should be considered exploratory, given the high rates of missing information. This reflects broader limitations in the quality and completeness of race and ethnicity information in Medicaid data. In regression models, unmeasured confounders related to individual characteristics such as health behaviors could have influenced our findings. Additionally, some chronic conditions such as COPD may mimic pneumonia symptoms and vice versa, potentially leading to misclassification in claims-based diagnoses. Such misclassification could affect our findings about the association of vulnerability and disease incidence if misclassification differs between more and less vulnerable areas. Lastly, there was some potential for spurious statistical significance, given the large number of statistical comparisons made. We chose not to adjust the type I error rate to avoid obscuring potentially important relationships in this analysis.

## Conclusion

Our analysis of Medicare and Medicaid claims data revealed disparities in pneumonia incidence between enrollees living in the most versus least socially vulnerable counties across the U.S. Observed disparities related to both individual demographic and clinical characteristics as well as county-level medical and non-medical SDOH, such as transportation availability, health care infrastructure, and access. These findings suggest that area-level vulnerability remains a significant and independent factor in pneumococcal disease burden, even after accounting for individual risk. Publicly available data on social vulnerability may be useful to identify root causes of disparities in pneumococcal disease and develop targeted interventions to reduce them. Tailoring prevention and outreach strategies to communities with high vulnerability could help address persistent inequities in pneumonia risk and strengthen the equity impact of public health planning.

## Supplementary information

Supplementary Information

Description of Additional Supplementary Files

Supplementary Data 1

Supplementary Data 2

Supplementary Data 3

Supplementary Data 4

Supplementary Data 5

Supplementary Data 6

Supplementary Data 7

Reporting Summary

## Data Availability

The data of Medicare and Medicaid beneficiaries are available under restricted access due to the requirements from CMS. Data access can be requested through ResDAC’s website (https://resdac.org/). Our data use agreement (DUA) prevents us from sharing data so cannot be made publicly available. According to the DUA, our group can only access data through the Virtual Data Resource Center (VRDC) with controlled access and cannot download the data. Aggregated source data for Figs. 1 through 4 are found in Supplementary Data 1–4. MHSVI data are available as of February 25, 2025 at https://minorityhealth.hhs.gov/minority-health-svi^16^.

## Code availability

The code used to analyze the data and generate findings of this study cannot be made publicly available, due to proprietary restrictions and DUA requirements.
