# Supplementary material for: Influence of area-level social vulnerability on all-cause pneumonia incidence among adult Medicare and Medicaid enrollees
Source: Commun Med (Lond). 2025 Nov 14;5:467. doi: 10.1038/s43856-025-01163-4 (PMC12618641; doi:10.1038/s43856-025-01163-4)
Supplement: Supplementary file 1 — Supplementary Information [file 43856_2025_1163_MOESM1_ESM.pdf]

Supplementary Information

Influence of area-level social vulnerability on all-cause pneumonia incidence among adult Medicare and Medicaid enrollees

Salini Mohanty[1], Priya Shanmugam[2], Saumya Chatrath[2], Kelsie Cassell[1], Nicole Cossrow[1], Peter C. Fiduccia[1], Esther Smith-Howell[1], Valina C. McGuinn[1], Jelena Zurovac[2], Constance Delannoy[2], Alyssa Evans[2], Michael Barna[2], Aparna Keshaviah[2], and Kristen A. Feemster[1]

[1] Merck & Co., Inc., Rahway, NJ, USA
[2] Mathematica, Inc., Princeton, NJ, USA

Table of Contents

Supplementary Table 1. Themes of Minority Health Social Vulnerability Index 4

Supplementary Table 2. States removed from Medicaid cohort analyses due to data quality issues 5

Supplementary Table 3. County-level urbanization classification using Rural-Urban Continuum Codes.. 5

Supplementary Table 4. Study cohort construction – number of beneficiaries at each stage of cohort creation 6

Supplementary Table 5. Unadjusted ACP incidence per 100,000 person-years among Medicare (2016–2019) and Medicaid beneficiaries (2017–2019), by age, risk of pneumococcal disease, and race and ethnicity.. 7

Supplementary Table 6. Moran's I to assess spatial autocorrelation in county-level disease incidence rates.. 8

Supplementary Table 7. Unadjusted ACP incidence per 100,000 person-years among Medicare (2016–2019) and Medicaid beneficiaries (2017–2019), by group and county vulnerability quintile, overall MHSVI 9

Supplementary Table 8. Difference in ACP incidence per 100,000 person-years among Medicare (2016–2019) and Medicaid enrollees (2017–2019) in the most and least socially vulnerable counties 10

Supplementary Table 9. Unadjusted ACP incidence per 100,000 person-years among Medicare (2016–2019) and Medicaid beneficiaries (2017–2019), by quintile and theme... 11

Supplementary Table 10. Unadjusted PP incidence per 100,000 person-years among Medicare (2016–2019) and Medicaid beneficiaries (2017–2019), by group and county vulnerability quintile, overall MHSVI 12

Supplementary Table 11. Unadjusted PP incidence per 100,000 person-years among Medicare (2016–2019) and Medicaid beneficiaries (2017–2019), by age, risk of pneumococcal disease, and race and ethnicity.. 13

Supplementary Table 12. Difference in PP incidence per 100,000 person-years among Medicare (2016–2019) and Medicaid enrollees (2017–2019) in the most and least socially vulnerable counties 14

Supplementary Table 13. Unadjusted PP incidence per 100,000 person-years among Medicare (2016–2019) and Medicaid beneficiaries (2017–2019), by quintile and theme... 15

Supplementary Table 14. Unadjusted IPD incidence per 100,000 person-years among Medicare (2016–2019) and Medicaid beneficiaries (2017–2019), by group and county vulnerability quintile, overall MHSVI 16

Supplementary Table 15. Unadjusted IPD incidence per 100,000 person-years among Medicare (2016–2019) and Medicaid beneficiaries (2017–2019), by age, risk of pneumococcal disease, and race and ethnicity 17

Supplementary Table 16. Difference in IPD incidence per 100,000 person-years among Medicare (2016–2019) and Medicaid enrollees (2017–2019) in the most and least socially vulnerable counties 18

Supplementary Table 17. Unadjusted IPD incidence per 100,000 person-years among Medicare (2016–2019) and Medicaid beneficiaries (2017–2019), by quintile and theme ... 19

Supplementary Figure 1: Unadjusted county-level PP incidence per 100,000 person-years 20

Supplementary Figure 2: Unadjusted PP incidence per 100,000 person-years by quintile of MHSVI and its themes 21

Supplementary Figure 3: Unadjusted county-level IPD incidence per 100,000 person-years 22

Supplementary Figure 4: Unadjusted IPD incidence per 100,000 person-years by quintile of MHSVI and its themes 23

Supplementary Table 1. Themes of Minority Health Social Vulnerability Index

Minority Health Social Vulnerability Index Themes					
Socioeconomic Status	Household Composition and Disability	Minority Status and Language	Housing Type and Transportation	Health Care Infrastructure and Access	Medical Vulnerability
Persons below 150% of Federal Poverty Line<br>Unemployed<br>Housing cost burden**<br>No high school diploma	≥65 Years<br>≤17 Years<br>Civilian with a disability<br>Single-parent households*	Hispanic or Latino<br>Black or African American<br>Asian<br>American Indian or Alaska Native<br>Native Hawaiian or Pacific Islander<br>Other Race<br>Two or More Races<br>Foreign language speakers: speaking English less than "very well"	Multi-unit structures*<br>Mobile homes*<br>Crowding (more people than rooms)**<br>No vehicle**<br>Persons living in group quarters	Hospitals*<br>Urgent care clinics*<br>Pharmacies*<br>Primary care physicians*<br>No health insurance	Cardiovascular disease<br>Chronic respiratory disease<br>Obesity<br>Diabetes<br>Internet access

Note: The themes and variables used to construct those themes are based on the 2018 data, released by the CDC in 2021.

*Measured at the county-level; **Measured at household level. All other variables measured at the individual-level.

Supplementary Table 2. States removed from Medicaid cohort analyses due to data quality issues

State	Data quality concerns in DQ Atlas in one or more years of measurement period (2017–2019)			Notes
	Identification of those for whom vaccine is recommended	Determining county of residence	Identifying disease episodes	
Florida	✓		✓	Low other services user rate
Maryland	✓		✓	Low diagnosis code capture in inpatient claims
New Hampshire	✓		✓	Low inpatient user rate
Rhode Island	✓	✓		Low ZIP code capture and low inpatient user rate
Tennessee	✓		✓	Low diagnosis code capture in inpatient claims
Vermont		✓		Low ZIP code capture

Note: We omitted states from the analyses for which the Data Quality (DQ) Atlas[1], a tool that reports Medicaid data quality, classified data fields critical for the analysis as “high concern” or “unusable” in at least one year within the study period (2017–2019): county of residence, procedure codes, diagnosis code, and proportion of service users. We also considered additional state-specific information, which led us to include one state—Georgia—where quality issues indicated in the DQ Atlas may have otherwise suggested to exclude it. DQ Atlas indicated that the proportion of service users in inpatient claims in Georgia was very high. This is because the inpatient (IP) file includes some other services (OT), but this would not impact our ability to identify disease episodes. Despite passing all data quality checks, we also excluded Arkansas and South Carolina from our analysis due to implausibly low disease incidence rates that were quite different from bordering states.

Despite passing all data quality checks, we also excluded Arkansas and South Carolina from our analysis due to implausibly low disease incidence rates that were quite different from bordering states. Colorado and New Mexico also had unexpectedly low disease incidence, but since this could be due to high vaccination rates, we kept them in our analysis.

Supplementary Table 3. County-level urbanization classification using Rural-Urban Continuum Codes

RUCC Description	Classification
Counties in metro areas of 1 million population or more	Urban
Counties in metro areas of 250,000 to 1 million population	Urban
Counties in metro areas of fewer than 250,000 population	Urban
Urban population of 20,000 or more, adjacent to a metro area	Suburban
Urban population of 20,000 or more, not adjacent to a metro area	Suburban
Urban population of 2,500 to 19,999, adjacent to a metro area	Suburban
Urban population of 2,500 to 19,999, not adjacent to a metro area	Suburban
Completely rural or less than 2,500 urban population, adjacent to a metro area	Rural
Completely rural or less than 2,500 urban population, not adjacent to a metro area	Rural

[1] https://www.medicaid.gov/dq-atlas/welcome

Supplementary Table 4. Study cohort construction – number of beneficiaries at each stage of cohort creation

Cohort Construction Step	Number of enrollees
Medicare (Aged ≥65 years)	
All beneficiaries in enrollment files, 2016–2019	71,529,841
After applying eligibility criteria	58,117,945
After dropping counties without MHSVI data	56,909,851
Medicaid (Aged 19–64 years)	
All beneficiaries in enrollment files, 2017–2019	58,883,885
After applying eligibility criteria	50,970,503
After dropping dual enrollees and long-term care beneficiaries	43,921,323
After dropping states with data quality concerns and counties without MHSVI data	36,579,701

Supplementary Table 5. Unadjusted ACP incidence per 100,000 person-years among Medicare (2016–2019) and Medicaid beneficiaries (2017–2019), by age, risk of pneumococcal disease, and race and ethnicity

	White, non-Hispanic	Black, non-Hispanic	Hispanic	Asian	Other
Medicare (Aged ≥65 years)					
Low risk					
65–74	333.5 (5,525,561)	196.2 (659,074)	218.2 (221,534)	299.3 (257,300)	273.9 (208,284)
75–84	521.6 (1,471,190)	307.5 (166,966)	270.5 (64,739)	457.7 (71,763)	447.7 (74,125)
85+	1,284.0 (649,617)	551.0 (82,776)	651.8 (32,065)	982.0 (34,369)	903.3 (25,034)
Moderate risk					
65–74	3,547.1 (7,020,927)	3,055.2 (1,128,404)	2,509.6 (312,614)	2,284.1 (321,152)	2,923.4 (263,972)
75–84	5,844.7 (4,167,823)	4,491.9 (578,797)	4,618.8 (139,712)	4,251.4 (182,229)	4,708.3 (181,043)
85+	13,486.4 (2,722,821)	9,615.4 (306,103)	12,630.3 (98,317)	11,496.3 (106,460)	10,479.8 (72,078)
High risk					
65–74	7,838.0 (10,121,059)	10,924.2 (1,106,703)	8,916.7 (244,281)	7,410.8 (220,643)	8,887.4 (219,268)
75–84	11,326.6 (9,377,568)	13,650.2 (817,086)	13,316.9 (164,400)	12,501.7 (179,552)	11,917.7 (223,836)
85+	19,178.3 (5,245,755)	19,927.1 (377,499)	24,560.1 (122,711)	24,222.9 (112,013)	19,958.4 (86,269)
Medicaid (Aged 19–64 years)					
Low risk					
19–49	937.0 (8,206,879)	687.2 (3,833,081)	521.9 (5,316,778)	444.4 (1,248,751)	887.0 (530,914)
50–64	1,767.6 (1,617,759)	1,636.8 (587,621)	1,024.2 (775,912)	776.3 (373,797)	2,039.3 (78,472)
Moderate risk					
19–49	4,130.3 (2,110,177)	3,385.4 (875,007)	3,189.1 (705,017)	2,083.1 (153,016)	4,046.2 (137,745)
50–64	7,739.8 (1,007,203)	6,624.0 (405,908)	4,524.4 (377,546)	2,624.8 (160,277)	7,807.8 (53,178)
High risk					
19–49	3,710.4 (311,787)	4,811.8 (215,487)	3,692.0 (139,981)	2,497.3 (31,276)	4,135.5 (18,142)
50–64	7,637.5 (163,575)	9,709.6 (71,297)	6,634.7 (58,499)	4,658.1 (23,483)	10,597.7 (5,597)

Note: Shown are disease incidence rates per 100,000 person-years, with numbers of beneficiaries in the group in parentheses.

Supplementary Table 6. Moran's I to assess spatial autocorrelation in county-level disease incidence rates

Measure	Coefficient	Z	Pr > |Z|
Medicare			
All-cause pneumonia	0.682	48.8	<.0001
Pneumococcal pneumonia	0.173	12.56	<.0001
Invasive pneumococcal disease	0.186	13.31	<.0001
Medicaid			
All-cause pneumonia	0.386	27.6	<.0001
Pneumococcal pneumonia	0.0538	4.14	<.0001
Invasive pneumococcal disease	0.0355	2.87	0.0041

Supplementary Table 7. Unadjusted ACP incidence per 100,000 person-years among Medicare (2016–2019) and Medicaid beneficiaries (2017–2019), by group and county vulnerability quintile, overall MHSVI

Group	N (in millions)	Overall ACP incidence rate	Unadjusted ACP incidence rate by MHSVI quintile					p-value (joint test for all quintiles)[a]
			Q1	Q2	Q3	Q4	Q5	
Medicare (Aged ≥65 years)								
Overall	56.9	7,910	7,073	7,750	7,831	7,928	8,203	<.0001
Age								
65–74	28.9	4,757	4,074	4,477	4,620	4,801	5,104	<.0001
75–84	17.9	8,605	7,739	8,370	8,479	8,632	8,973	<.0001
85+	10.1	16,003	14,675	16,062	16,069	15,918	16,252	<.0001
Risk of pneumococcal disease								
Low risk	9.9	416	518	455	449	406	357	<.0001
Moderate risk	17.9	5,635	5,638	5,819	5,747	5,688	5,426	<.0001
High risk	29.1	11,571	10,079	11,068	11,357	11,427	12,436	<.0001
Race and ethnicity								
White, non-Hispanic	46.3	8,143	7,179	7,950	8,102	8,215	8,489	<.0001
Black, non-Hispanic	5.2	7,680	6,967	7,524	7,504	7,504	7,867	<.0001
Hispanic	1.4	7,320	5,618	6,198	6,880	6,724	7,683	<.0001
Asian	1.5	6,452	5,163	5,647	6,433	5,947	7,020	<.0001
Other	1.4	6,576	7,163	6,008	6,124	6,456	7,121	<.0001
Medicaid (Aged 19–64 years)								
Overall	36.6	1,819	2,095	2,067	1,855	1,926	1,650	<.0001
Age								
19–49	29.4	1,362	1,653	1,603	1,407	1,439	1,204	<.0001
50–64	7.2	3,597	3,807	3,849	3,545	3,817	3,377	<.0001
Risk of pneumococcal disease								
Low risk	28.0	848	1,049	988	885	891	755	<.0001
Moderate risk	7.3	4,645	4,840	4,802	4,611	4,694	4,558	<.0001
High risk	1.3	5,137	4,277	4,755	4,944	5,004	5,510	<.0001
Race and ethnicity								
White, non-Hispanic	13.4	2,273	2,192	2,316	2,282	2,402	2,145	<.0001
Black, non-Hispanic	6.0	1,916	1,742	1,834	1,898	1,969	1,902	<.0001
Hispanic	7.4	1,188	1,704	1,571	1,247	1,221	1,132	<.0001
Asian	2.0	918	1,097	948	861	938	934	<.0001
Other	0.8	2,189	2,333	2,169	2,311	2,333	2,039	<.0001

Note: Incidence rates are per 100,000 person-years.

ACP = All-cause pneumonia; MHSVI = Minority Health Social Vulnerability Index, Q1 = least vulnerabe quintile; Q5 = most vulnerable quintile.

[a] The joint test evaluates whether the regression coefficients for all quintile categories are statistically significant from zero together, which assesses whether vulnerability (as divided into quintiles) affects disease incidence overall.

Supplementary Table 8. Difference in ACP incidence per 100,000 person-years among Medicare (2016–2019) and Medicaid enrollees (2017–2019) in the most and least socially vulnerable counties

Demographic group	Unadjusted results						Regression-adjusted results					
	N	Incidence Difference (Q5-Q1)	IRR (Q5/Q1)	95% CI around IRR	IRR *p*-value	AIC	N	Incidence Difference (Q5-Q1)	IRR (Q5/Q1)	95% CI around IRR	IRR *p*-value	AIC
Medicare (Aged ≥65 years)												
Overall	56,909,851	1,129.64	1.16	(1.157, 1.162)	<.0001	91,123,930	56,909,618	37.65	1.12	(1.117, 1.122)	<.0001	81,553,643
Age												
65–74	28,856,668	1,029.93	1.25	(1.247, 1.258)	<.0001	31,045,120	28,856,668	36.10	1.13	(1.123, 1.133)	<.0001	27,811,495
75–84	17,948,838	1,233.61	1.16	(1.155, 1.164)	<.0001	32,559,778	17,948,838	54.29	1.12	(1.117, 1.126)	<.0001	30,473,479
85+	10,104,112	1,576.92	1.11	(1.103, 1.112)	<.0001	24,054,515	10,104,112	114.53	1.10	(1.097, 1.106)	<.0001	23,058,655
Risk of pneumococcal disease												
Low risk	9,859,367	-160.83	0.69	(0.674, 0.705)	<.0001	1,435,129	9,859,319	-72.70	0.74	(0.723, 0.758)	<.0001	1,390,247
Moderate risk	17,945,773	-211.90	0.96	(0.958, 0.967)	<.0001	22,002,519	17,945,748	113.94	1.04	(1.031, 1.041)	<.0001	20,726,985
High risk	29,104,711	2,357.39	1.23	(1.231, 1.237)	<.0001	61,795,901	29,104,551	1,070.74	1.16	(1.145, 1.160)	<.0001	59,248,613
Race and ethnicity												
White, non-Hispanic	46,302,403	1,310.65	1.18	(1.180, 1.185)	<.0001	75,588,592	46,302,284	38.10	1.12	(1.114, 1.120)	<.0001	68,058,613
Black, non-Hispanic	5,223,475	900.31	1.13	(1.104, 1.155)	<.0001	8,244,714	5,223,404	11.69	1.06	(1.033, 1.080)	<.0001	7,240,973
Hispanic	1,400,384	2,065.35	1.37	(1.287, 1.453)	<.0001	2,105,770	1,400,368	19.56	1.12	(1.049, 1.185)	0.0004	1,794,608
Asian	1,485,490	1,857.50	1.36	(1.303, 1.419)	<.0001	2,090,566	1,485,480	34.22	1.13	(1.081, 1.178)	<.0001	1,769,186
Other	1,353,925	-41.97	0.99	(0.970, 1.019)	0.6430	1,939,931	1,353,908	32.25	1.12	(1.095, 1.152)	<.0001	1,686,189
Medicaid (Aged 19–64 years)												
Overall	36,579,701	-444.34	0.79	(0.780, 0.795)	<.0001	12,448,681	36,577,700	16.23	1.02	(1.009, 1.029)	0.0002	11,303,773
Age												
19–49	29,383,244	-449.37	0.73	(0.719, 0.737)	<.0001	7,763,986	29,381,308	-26.82	0.97	(0.957, 0.982)	<.0001	7,218,548
50–64	7,196,457	-430.87	0.89	(0.873, 0.901)	<.0001	4,396,338	7,196,392	160.09	1.11	(1.088, 1.124)	<.0001	4,081,338
Risk of pneumococcal disease												
Low risk	27,973,467	-294.08	0.72	(0.708, 0.731)	<.0001	4,899,470	27,971,534	-83.16	0.91	(0.891, 0.921)	<.0001	4,830,863
Moderate risk	7,330,617	-282.36	0.94	(0.930, 0.954)	<.0001	5,512,983	7,330,565	227.72	1.06	(1.045, 1.073)	<.0001	5,427,424
High risk	1,275,617	1,233.45	1.29	(1.247, 1.332)	<.0001	1,053,202	1,275,601	1,074.92	1.24	(1.196, 1.280)	<.0001	1,028,081
Race and ethnicity												
White, non-Hispanic	13,417,380	-46.54	0.98	(0.967, 0.991)	0.0004	5,423,949	13,417,270	36.77	1.04	(1.032, 1.057)	<.0001	4,981,715
Black, non-Hispanic	5,988,401	159.99	1.09	(1.034, 1.153)	0.0015	2,153,025	5,988,379	19.30	1.03	(0.972, 1.084)	0.3435	1,945,143
Hispanic	7,373,733	-572.11	0.66	(0.641, 0.688)	<.0001	1,791,976	7,373,713	-65.71	0.90	(0.862, 0.931)	<.0001	1,632,034
Asian	1,990,600	-162.81	0.85	(0.766, 0.947)	0.0029	399,223	1,990,595	-59.57	0.89	(0.801, 0.997)	0.0443	374,463
Other	824,048	-293.38	0.87	(0.820, 0.932)	<.0001	324,554	824,044	-1.77	1.00	(0.936, 1.065)	0.9519	296,775

Note: Incidence rates are per 100,000 person-years.

ACP = all-cause pneumonia; AIC = Akaike information criterion; CI = confidence interval; IRR = incidence rate ratio; MHSVI = Minority Health Social Vulnerability Index; Q1 = least vulnerabe quintile; Q5 = most vulnerable quintile.

Supplementary Table 9. Unadjusted ACP incidence per 100,000 person-years among Medicare (2016–2019) and Medicaid beneficiaries (2017–2019), by quintile and theme

Theme	Unadjusted ACP incidence by MHSVI Quintile					IRR (Q5/Q1)	IRR *p*-value
	Q1	Q2	Q3	Q4	Q5		
Medicare (Aged ≥65 years)							
Overall MHSVI	7,073	7,750	7,831	7,928	8,203	1.16	<.0001
Socioeconomic Status	7,277	7,687	8,035	8,428	8,974	1.23	<.0001
Household Composition and Disability	7,426	7,777	8,172	8,616	9,320	1.25	<.0001
Minority Status and Language	9,448	9,150	8,532	8,241	7,639	0.81	<.0001
Housing Type and Transportation	7,501	7,922	8,013	7,977	7,904	1.05	<.0001
Health Care Infrastructure and Access	7,941	7,643	7,954	8,344	8,600	1.08	<.0001
Medical Vulnerability	7,318	7,837	8,307	9,266	10,022	1.37	<.0001
Medicaid (Aged 19–64 years)							
Overall MHSVI	2,095	2,067	1,855	1,926	1,650	0.79	<0.0001
Socioeconomic Status	1,832	1,891	1,895	1,634	1,843	1.01	0.165
Household Composition and Disability	1,475	2,044	2,015	2,060	2,350	1.59	<0.0001
Minority Status and Language	2,621	2,552	2,477	2,275	1,658	0.63	<0.0001
Housing Type and Transportation	2,071	2,153	1,977	2,019	1,537	0.74	<0.0001
Health Care Infrastructure and Access	1,975	1,752	1,624	2,081	2,302	1.17	<0.0001
Medical Vulnerability	1,516	1,975	2,147	2,437	2,576	1.70	<0.0001

Note: Incidence rates are per 100,000 person-years.

ACP = All-cause pneumonia; IRR = incidence rate ratio; MHSVI = Minority Health Social Vulnerability Index; Q1 = least vulnerabe quintile; Q5 = most vulnerable quintile.

Supplementary Table 10. Unadjusted PP incidence per 100,000 person-years among Medicare (2016–2019) and Medicaid beneficiaries (2017–2019), by group and county vulnerability quintile, overall MHSVI

Group	N (in millions)	Overall PP incidence rate	Unadjusted PP incidence rate by MHSVI quintile					p-value (joint test for all quintiles)[a]
			Q1	Q2	Q3	Q4	Q5	
Medicare (Aged ≥65 years)								
Overall	56.9	42.01	41.64	40.35	40.36	39.86	45.93	<.0001
Age								
65–74	28.9	26.94	24.88	24.32	25.69	25.53	30.78	<.0001
75–84	17.9	46.03	46.03	43.68	43.34	44.44	50.58	<.0001
85+	10.1	79.20	82.72	80.47	77.92	73.63	83.34	<.0001
Risk of pneumococcal disease								
Low risk	9.9	1.91	1.97	2.00	2.03	1.89	1.77	.7634
Moderate risk	17.9	31.75	34.12	30.76	30.84	29.30	34.29	<.0001
High risk	29.1	60.46	59.12	57.49	57.91	57.09	67.19	<.0001
Race and ethnicity								
White, non-Hispanic	46.3	42.98	42.15	41.38	41.68	41.68	46.57	<.0001
Black, non-Hispanic	5.2	36.56	24.71	37.86	37.87	33.33	38.04	<.0001
Hispanic	1.4	45.24	148.95	40.42	37.96	37.52	48.81	<.0001
Asian	1.5	45.87	28.72	34.51	32.88	29.52	70.24	<.0001
Other	1.4	40.25	52.52	29.60	33.07	37.60	49.68	<.0001
Medicaid (Aged 19–64 years)								
Overall	36.6	9.60	10.27	11.22	10.30	10.22	8.40	<.0001
Age								
19–49	29.4	5.83	7.33	6.64	6.36	6.15	5.03	<.0001
50–64	7.2	24.29	21.67	28.80	25.15	26.05	21.44	<.0001
Risk of pneumococcal disease								
Low risk	28.0	3.58	3.41	4.42	3.43	3.97	3.24	<.0001
Moderate risk	7.3	27.03	28.76	28.35	30.57	26.44	24.87	<.0001
High risk	1.3	30.53	21.59	29.06	27.60	31.88	32.30	.2023
Race and ethnicity								
White, non-Hispanic	13.4	12.57	10.52	12.60	13.72	13.57	11.20	<.0001
Black, non-Hispanic	6.0	9.97	5.26	10.34	10.18	10.18	9.78	.6203
Hispanic	7.4	5.41	6.48	7.44	5.99	5.49	5.10	.1271
Asian	2.0	4.25	6.27	5.03	2.75	4.39	4.98	.0470
Other	0.8	12.35	16.25	10.69	13.28	12.35	11.83	.8728

Note: Incidence rates are per 100,000 beneficiary-years. MHSVI = Minority Health Social Vulnerability Index; PP = Pneumococcal pneumonia; Q1 is least vulnerabe quintile; Q5 is most vulnerable quintile.

[a] The joint test evaluates whether the regression coefficients for all quintile categories are statistically significant from zero together, which assesses whether vulnerability (as divided into quintiles) affects disease incidence overall.

Supplementary Table 11. Unadjusted PP incidence per 100,000 person-years among Medicare (2016–2019) and Medicaid beneficiaries (2017–2019), by age, risk of pneumococcal disease, and race and ethnicity

Group	Unadjusted PP incidence (N)				
	White, non-Hispanic	Black, non-Hispanic	Hispanic	Asian	Other
Medicare (Aged ≥65 years)					
Low risk					
65–74	1.5 (5,525,561)	0.9 (659,074)	0.6 (221,534)	1.9 (257,300)	1.2 (208,284)
75–84	2.5 (1,471,190)	2.4 (166,966)	1.7 (64,739)	3.4 (71,763)	3.2 (74,125)
85+	5.7 (649,617)	1.9 (82,776)	0.0 (32,065)	4.8 (34,369)	0.0 (25,034)
Moderate risk					
65–74	21.6 (7,020,927)	17.6 (1,128,404)	17.2 (312,614)	28.1 (321,152)	23.7 (263,972)
75–84	32.2 (4,167,823)	24.2 (578,797)	39.2 (139,712)	39.8 (182,229)	27.7 (181,043)
85+	67.3 (2,722,821)	44.7 (306,103)	76.3 (98,317)	79.8 (106,460)	69.3 (72,078)
High risk					
65–74	43.4 (10,121,059)	53.2 (1,106,703)	48.3 (244,281)	53.8 (220,643)	55.9 (219,268)
75–84	60.1 (9,377,568)	61.4 (817,086)	81.0 (164,400)	81.5 (179,552)	66.7 (223,836)
85+	93.7 (5,245,755)	85.8 (377,499)	152.8 (122,711)	132.4 (112,013)	118.3 (86,269)
Medicaid (Aged 19–64 years)					
Low risk					
19–49	3.5 (8,206,879)	2.6 (3,833,081)	1.5 (5,316,778)	1.7 (1,248,751)	3.1 (530,914)
50–64	10.5 (1,617,759)	10.5 (587,621)	5.3 (775,912)	3.4 (373,797)	9.9 (78,472)
Moderate risk					
19–49	19.2 (2,110,177)	13.7 (875,007)	16.7 (705,017)	9.2 (153,016)	25.5 (137,745)
50–64	59.7 (1,007,203)	43.0 (405,908)	24.9 (377,546)	17.6 (160,277)	50.9 (53,178)
High risk					
19–49	18.1 (311,787)	26.1 (215,487)	13.8 (139,981)	7.2 (31,276)	34.7 (18,142)
50–64	60.3 (163,575)	76.6 (71,297)	44.4 (58,499)	15.3 (23,483)	66.4 (5,597)

Note: Shown are disease incidence rates per 100,000 person-years, with numbers of beneficiaries in the group in parentheses.

DI = disease incidence rate; PP = Pneumococcal pneumonia.

Supplementary Table 12. Difference in PP incidence per 100,000 person-years among Medicare (2016–2019) and Medicaid enrollees (2017–2019) in the most and least socially vulnerable counties

Group	Unadjusted results						Regression-adjusted results					
	N	Incidence Difference (Q5-Q1)	IRR (Q5/Q1)	95% CI around IRR	IRR *p*-value	AIC	N	Incidence Difference (Q5-Q1)	IRR (Q5/Q1)	95% CI around IRR	IRR *p*-value	AIC
Medicare (Aged ≥65 years)												
Overall	56,909,851	4.29	1.10	(1.070, 1.138)	<.0001	1,274,977	56,909,618	0.14	1.09	(1.055, 1.124)	<.0001	1,227,715
Age												
65–74	28,856,668	5.90	1.24	(1.169, 1.309)	<.0001	424,484	28,856,668	0.18	1.14	(1.079, 1.212)	<.0001	405,943
75–84	17,948,838	4.55	1.10	(1.045, 1.156)	0.0002	469,779	17,948,838	0.24	1.10	(1.043, 1.157)	0.0004	459,103
85+	10,104,112	0.62	1.01	(0.954, 1.064)	0.7868	366,204	10,104,112	0.06	1.01	(0.956, 1.071)	0.6884	361,354
Risk of pneumococcal disease												
Low risk	9,859,367	-0.20	0.90	(0.629, 1.272)	0.5351	12,940	9,859,319	-0.18	0.85	(0.592, 1.224)	0.3849	12,755
Moderate risk	17,945,773	0.17	1.00	(0.945, 1.069)	0.8760	312,874	17,945,748	1.49	1.07	(1.006, 1.143)	0.0323	307,292
High risk	29,104,711	8.07	1.14	(1.096, 1.178)	<.0001	918,792	29,104,551	4.03	1.10	(1.060, 1.142)	<.0001	907,063
Race and ethnicity												
White, non-Hispanic	46,302,403	4.42	1.10	(1.070, 1.141)	<.0001	1,060,139	46,302,284	0.12	1.08	(1.040, 1.111)	<.0001	1,023,176
Black, non-Hispanic	5,223,475	13.33	1.54	(1.061, 2.233)	0.0230	102,406	5,223,404	0.36	1.42	(0.977, 2.057)	0.0660	98,228
Hispanic	1,400,384	-100.14	0.33	(0.225, 0.476)	<.0001	32,977	1,400,368	-1.52	0.27	(0.184, 0.396)	<.0001	31,102
Asian	1,485,490	41.52	2.45	(1.385, 4.318)	0.0020	35,787	1,485,480	1.26	1.80	(1.019, 3.183)	0.0431	33,988
Other	1,353,925	-2.84	0.95	(0.707, 1.265)	0.7081	29,982	1,353,908	0.22	1.18	(0.876, 1.592)	0.2754	28,328
Medicaid (Aged 19–64 years)												
Overall	36,579,701	-1.87	0.82	(0.714, 0.938)	0.0041	140,901	36,577,700	0.34	1.11	(0.965, 1.277)	0.1443	131,626
Age												
19-49	29,383,244	-2.30	0.69	(0.572, 0.824)	<.0001	70,790	29,381,308	-0.25	0.93	(0.772, 1.124)	0.4580	67,621
50-64	7,196,457	-0.23	0.99	(0.805, 1.217)	0.9196	66,626	7,196,392	2.62	1.35	(1.090, 1.664)	0.0058	63,946
Risk of pneumococcal disease												
Low risk	27,973,467	-0.18	0.95	(0.718, 1.252)	0.7083	42,777	27,971,534	0.63	1.21	(0.914, 1.615)	0.1808	42,042
Moderate risk	7,330,617	-3.89	0.86	(0.731, 1.023)	0.0896	76,446	7,330,565	0.38	1.02	(0.859, 1.212)	0.8180	75,197
High risk	1,275,617	10.71	1.50	(0.943, 2.373)	0.0871	14,611	1,275,601	9.27	1.44	(0.897, 2.310)	0.1310	14,283
Race and ethnicity												
White, non-Hispanic	13,417,380	0.68	1.06	(0.896, 1.264)	0.4770	66,229	13,417,270	0.31	1.11	(0.931, 1.319)	0.2471	62,173
Black, non-Hispanic	5,988,401	4.52	1.86	(0.696, 4.970)	0.2163	24,229	5,988,379	1.17	1.62	(0.604, 4.327)	0.3388	22,668
Hispanic	7,373,733	-1.37	0.79	(0.445, 1.397)	0.4147	16,927	7,373,713	0.37	1.24	(0.676, 2.269)	0.4889	15,806
Asian	1,990,600	-1.29	0.79	(0.195, 3.226)	0.7471	3,708	1,990,595	-0.85	0.73	(0.175, 3.091)	0.6741	3,548
Other	824,048	-4.42	0.73	(0.337, 1.572)	0.4191	3,934	824,044	0.31	1.11	(0.505, 2.419)	0.8014	3,653

Note: Shown are disease incidence rates per 100,000 person-years.

AIC = Akaike information criterion; CI = confidence interval; IRR = incidence rate ratio; MHSVI = Minority Health Social Vulnerability Index; PP = pneumococcal pneumonia; Q1 = least vulnerabe quintile; Q5 = most vulnerable quintile.

Supplementary Table 13. Unadjusted PP incidence per 100,000 person-years among Medicare (2016–2019) and Medicaid beneficiaries (2017–2019), by quintile and theme

Theme	Unadjusted PP incidence MHSVI Quintile					IRR (Q5/Q1)	IRR *p*-value
	Q1	Q2	Q3	Q4	Q5		
Medicare (Aged ≥65 years)							
Overall MHSVI	41.6	40.4	40.4	39.9	45.9	1.10	<.0001
Socioeconomic Status	36.5	39.8	42.5	47.9	51.1	1.40	<.0001
Household Composition and Disability	36.6	39.9	45.1	53.8	51.8	1.42	<.0001
Minority Status and Language	50.5	50.9	48.0	47.3	39.4	0.78	<.0001
Housing Type and Transportation	39.8	41.8	39.3	44.1	42.5	1.07	0.0008
Health Care Infrastructure and Access	43.6	37.1	43.6	47.9	47.3	1.09	<.0001
Medical Vulnerability	37.9	42.7	45.5	51.4	51.0	1.35	<.0001
Medicaid (Aged 19–64 years)							
Overall MHSVI	10.3	11.2	10.3	10.2	8.4	0.82	0.020
Socioeconomic Status	8.6	9.7	11.3	8.4	9.4	1.09	0.101
Household Composition and Disability	7.0	10.6	11.7	11.6	13.8	1.97	<.0001
Minority Status and Language	13.7	17.4	13.1	12.9	8.4	0.61	<.0001
Housing Type and Transportation	10.5	10.9	10.3	11.1	8.0	0.76	0.015
Health Care Infrastructure and Access	11.1	9.1	8.0	11.2	12.8	1.15	0.079
Medical Vulnerability	7.4	11.1	12.5	13.8	12.6	1.70	<.0001

Note: Incidence rates are per 100,000 person-years.

IRR = incidence rate ratio; MHSVI = Minority Health Social Vulnerability Index; PP = Pneumococcal pneumonia; Q1 is least vulnerabe quintile; Q5 is most vulnerable quintile.

Supplementary Table 14. Unadjusted IPD incidence per 100,000 person-years among Medicare (2016–2019) and Medicaid beneficiaries (2017–2019), by group and county vulnerability quintile, overall MHSVI

Group	N (in millions)	Overall IPD incidence rate	Unadjusted IPD incidence rate by MHSVI quintile					p-value (joint test for all quintiles)[a]
			Q1	Q2	Q3	Q4	Q5	
Medicare (Aged ≥65 years)								
Overall	56.9	41.30	38.76	38.96	40.39	42.37	42.88	<.0001
Age								
65–74	28.9	29.45	26.29	26.36	28.01	31.04	31.51	<.0001
75–84	17.9	44.36	40.54	41.48	44.25	45.69	45.70	<.0001
85+	10.1	70.76	72.42	70.67	69.36	69.68	72.42	.1067
Risk of pneumococcal disease								
Low risk	9.9	1.12	1.14	1.24	1.02	1.05	1.16	.7777
Moderate risk	17.9	26.56	26.24	24.73	26.80	28.33	26.02	<.0001
High risk	29.1	62.44	58.58	58.48	60.64	62.58	66.73	<.0001
Race and ethnicity								
White, non-Hispanic	46.3	40.91	38.97	39.20	40.84	41.77	41.84	<.0001
Black, non-Hispanic	5.2	51.29	45.89	47.41	47.30	52.93	52.14	.0026
Hispanic	1.4	42.42	21.28	44.00	41.23	42.49	42.68	.5922
Asian	1.5	33.80	31.12	29.91	35.77	35.12	32.48	.2128
Other	1.4	42.01	70.02	36.16	37.15	42.18	45.82	<.0001
Medicaid (Aged 19–64 years)								
Overall	36.6	14.13	14.29	13.99	13.67	16.51	12.95	<.0001
Age								
19–49	29.4	8.64	8.38	9.07	8.26	10.03	7.87	<.0001
50–64	7.2	35.49	37.24	32.87	34.11	41.70	32.61	<.0001
Risk of pneumococcal disease								
Low	28.0	5.80	5.35	5.93	5.65	6.75	5.33	<.0001
Moderate	7.3	35.98	33.17	33.27	34.65	39.97	35.02	<.0001
High	1.3	56.48	60.23	41.52	50.33	61.07	60.63	<.0001
Race and ethnicity								
White, non-Hispanic	13.4	16.67	14.63	14.73	16.41	19.37	16.08	<.0001
Black, non-Hispanic	6.0	16.91	15.78	14.26	16.84	18.66	16.27	.0177
Hispanic	7.4	8.42	10.80	9.11	7.99	10.40	7.87	.0021
Asian	2.0	4.30	15.67	4.31	5.40	3.27	4.01	.0184
Other	0.8	31.60	18.57	30.73	23.74	44.59	28.45	<.0001

Note: Incidence rates are per 100,000 person-years.

IPD = Invasive pneumococcal disease; MHSVI = Minority Health Social Vulnerability Index, Q1 is least vulnerabe quintile; Q5 is most vulnerable quintile.

[a] The joint test evaluates whether the regression coefficients for all quintile categories are statistically significant from zero together, which assesses whether vulnerability (as divided into quintiles) affects disease incidence overall.

Supplementary Table 15. Unadjusted IPD incidence per 100,000 person-years among Medicare (2016–2019) and Medicaid beneficiaries (2017–2019), by age, risk of pneumococcal disease, and race and ethnicity

Group	Unadjusted IPD incidence (N)				
	White, non-Hispanic	Black, non-Hispanic	Hispanic	Asian	Other
Medicare (Aged ≥65 years)					
Low risk					
65–74	0.7 (5,525,561)	0.7 (659,074)	2.9 (221,534)	0.8 (257,300)	1.2 (208,284)
75–84	1.2 (1,471,190)	1.6 (166,966)	0.8 (64,739)	0.4 (71,763)	2.1 (74,125)
85+	3.3 (649,617)	2.6 (82,776)	9.4 (32,065)	1.9 (34,369)	2.4 (25,034)
Moderate risk					
65–74	18.5 (7,020,927)	20.1 (1,128,404)	14.4 (312,614)	11.5 (321,152)	19.9 (263,972)
75–84	26.8 (4,167,823)	29.1 (578,797)	21.0 (139,712)	15.3 (182,229)	24.9 (181,043)
85+	53.2 (2,722,821)	52.7 (306,103)	61.3 (98,317)	52.3 (106,460)	60.6 (72,078)
High risk					
65–74	49.4 (10,121,059)	84.6 (1,106,703)	59.9 (244,281)	44.3 (220,643)	72.4 (219,268)
75–84	57.6 (9,377,568)	89.8 (817,086)	83.9 (164,400)	74.6 (179,552)	71.4 (223,836)
85+	84.1 (5,245,755)	113.5 (377,499)	128.9 (122,711)	125.6 (112,013)	116.5 (86,269)
Medicaid (Aged 19–64 years)					
Low risk					
19–49	4.4 (8,206,879)	4.8 (3,833,081)	2.6 (5,316,778)	1.4 (1,248,751)	10.4 (530,914)
50–64	18.8 (1,617,759)	23.0 (587,621)	9.0 (775,912)	3.5 (373,797)	23.3 (78,472)
Moderate risk					
19–49	25.3 (2,110,177)	22.3 (875,007)	18.3 (705,017)	11.2 (153,016)	56.2 (137,745)
50–64	71.2 (1,007,203)	57.3 (405,908)	41.6 (377,546)	11.4 (160,277)	139.5 (53,178)
High risk					
19–49	33.9 (311,787)	49.6 (215,487)	37.9 (139,981)	18.6 (31,276)	69.5 (18,142)
50–64	80.8 (163,575)	156.2 (71,297)	76.6 (58,499)	45.9 (23,483)	199.2 (5,597)

Note: Shown are disease incidence rates per 100,000 person-years, with numbers of beneficiaries in the group in parentheses.
DI = disease incidence rate; IPD = Invasive pneumococcal disease.

Supplementary Table 16. Difference in IPD incidence per 100,000 person-years among Medicare (2016–2019) and Medicaid enrollees (2017–2019) in the most and least socially vulnerable counties

Group	Unadjusted results						Regression-adjusted results					
	N	Incidence Difference (Q5-Q1)	IRR (Q5/Q1)	95% CI around IRR	IRR *p*-value	AIC	N	Incidence Difference (Q5-Q1)	IRR (Q5/Q1)	95% CI around IRR	IRR *p*-value	AIC
Medicare (Aged ≥65 years)												
Overall	56,909,851	4.12	1.11	(1.071, 1.142)	<.0001	1,264,058	56,909,618	0.00	1.00	(0.969, 1.035)	0.9451	1,211,142
Age												
65–74	28,856,668	5.22	1.20	(1.134, 1.266)	<.0001	461,780	28,856,668	0.00	1.00	(0.945, 1.059)	0.9836	435,879
75–84	17,948,838	5.16	1.13	(1.068, 1.189)	<.0001	458,418	17,948,838	0.04	1.04	(0.981, 1.095)	0.2029	444,874
85+	10,104,112	0.00	1.00	(0.943, 1.060)	0.9994	334,568	10,104,112	-0.17	0.95	(0.898, 1.014)	0.1328	328,968
Risk of pneumococcal disease												
Low risk	9,859,367	0.02	1.02	(0.642, 1.604)	0.9510	8,127	9,859,319	-0.12	0.83	(0.511, 1.338)	0.4382	7,978
Moderate risk	17,945,773	-0.22	0.99	(0.925, 1.064)	0.8195	270,551	17,945,748	0.02	1.00	(0.931, 1.077)	0.9769	264,982
High risk	29,104,711	8.15	1.14	(1.099, 1.181)	<.0001	949,553	29,104,551	0.28	1.01	(0.969, 1.044)	0.7599	937,153
Race and ethnicity												
White, non-Hispanic	46,302,403	2.87	1.07	(1.038, 1.110)	<.0001	1,020,580	46,302,284	0.01	1.01	(0.972, 1.041)	0.7536	980,676
Black, non-Hispanic	5,223,475	6.25	1.14	(0.864, 1.493)	0.3600	139,252	5,223,404	0.03	1.03	(0.782, 1.351)	0.8464	132,349
Hispanic	1,400,384	21.40	2.01	(0.752, 5.353)	0.1646	31,364	1,400,368	0.92	1.66	(0.617, 4.444)	0.3167	29,634
Asian	1,485,490	1.36	1.04	(0.603, 1.809)	0.8780	28,157	1,485,480	-0.10	0.86	(0.495, 1.499)	0.5967	26,242
Other	1,353,925	-24.20	0.65	(0.507, 0.844)	0.0011	31,143	1,353,908	-0.26	0.83	(0.640, 1.080)	0.1661	29,256
Medicaid (Aged 19–64 years)												
Overall	36,579,701	-1.34	0.91	(0.807, 1.017)	0.095	199,091	36,577,700	0.52	1.00	(0.966, 1.032)	0.1549	185,532
Age												
19–49	29,383,244	-0.51	0.94	(0.793, 1.112)	0.468	101,133	29,381,308	0.78	1.15	(0.970, 1.370)	0.1071	95,722
50–64	7,196,457	-4.63	0.88	(0.747, 1.026)	0.1	92,924	7,196,392	0.83	1.04	(0.886, 1.226)	0.6167	89,607
Risk of pneumococcal disease												
Low risk	27,973,467	-0.02	1.00	(0.798, 1.244)	0.976	66,791	27,971,534	0.78	1.17	(0.931, 1.466)	0.1798	64,861
Moderate risk	7,330,617	1.85	1.06	(0.904, 1.233)	0.492	97,871	7,330,565	3.88	1.14	(0.975, 1.339)	0.0988	96,045
High risk	1,275,617	0.40	1.01	(0.761, 1.331)	0.963	24,959	1,275,601	-17.3	0.79	(0.588, 1.049)	0.1014	24,274
Race and ethnicity												
White, non-Hispanic	13,417,380	1.45	1.10	(0.950, 1.271)	0.203	84,391	13,417,270	0.65	1.12	(0.970, 1.302)	0.1210	79,279
Black, non-Hispanic	5,988,401	0.49	1.03	(0.583, 1.821)	0.917	38,803	5,988,379	-1.4	0.84	(0.473, 1.479)	0.5386	36,286
Hispanic	7,373,733	-2.93	0.73	(0.468, 1.136)	0.163	25,384	7,373,713	0.15	1.04	(0.645, 1.683)	0.8659	23,567
Asian	1,990,600	-11.67	0.26	(0.103, 0.633)	0.003	3,813	1,990,595	-7.97	0.22	(0.084, 0.589)	0.0025	3,586
Other	824,048	9.88	1.53	(0.756, 3.103)	0.236	9,076	824,044	5.32	1.78	(0.875, 3.624)	0.1112	8,484

Note: Shown are incidence rates per 100,000 person-years.

AIC = Akaike information criterion; CI = confidence interval; IPD = Invasive pneumococcal disease; IRR = incidence rate ratio; MHSVI = Minority Health Social Vulnerability Index; Q1 = least vulnerabe quintile; Q5 = most vulnerable quintile.

Supplementary Table 17. Unadjusted IPD incidence per 100,000 person-years among Medicare (2016–2019) and Medicaid beneficiaries (2017–2019), by quintile and theme

Theme	Unadjusted IPD incidence MHSVI Quintile					IRR (Q5/Q1)	IRR *p*-value
	Q1	Q2	Q3	Q4	Q5		
Medicare (Aged ≥65 years)							
Overall MHSVI	38.8	39.0	40.4	42.4	42.9	1.11	0.0006
Socioeconomic Status	36.5	40.8	43.6	43.6	45.0	1.23	<.0001
Household Composition and Disability	38.0	40.1	45.2	45.7	47.8	1.26	<.0001
Minority Status and Language	48.4	47.7	45.2	44.8	39.5	0.82	<.0001
Housing Type and Transportation	38.5	39.1	40.9	42.6	42.2	1.10	<.0001
Health Care Infrastructure and Access	42.9	40.1	38.7	44.6	47.8	1.11	<.0001
Medical Vulnerability	36.8	42.9	46.5	49.2	49.0	1.33	<.0001
Medicaid (Aged 19–64 years)							
Overall MHSVI	14.3	14.0	13.7	16.5	13.0	0.91	0.251
Socioeconomic Status	13.9	15.1	15.7	12.1	13.3	0.96	0.282
Household Composition and Disability	11.2	16.8	14.7	16.3	19.0	1.70	<.0001
Minority Status and Language	16.2	17.6	15.4	17.6	13.4	0.83	0.005
Housing Type and Transportation	15.2	14.8	14.2	16.0	12.6	0.83	0.005
Health Care Infrastructure and Access	15.0	15.0	11.6	15.3	19.0	1.27	<.0001
Medical Vulnerability	12.1	15.2	18.0	17.4	17.1	1.41	<.0001

Note: Incidence rates are per 100,000 person-years.

IPD = Invasive pneumococcal disease; MHSVI = Minority Health Social Vulnerability Index, Q1 is least vulnerabe quintile; Q5 is most vulnerable quintile.

a. Medicare FFS and MA beneficiaries (2016–2019)

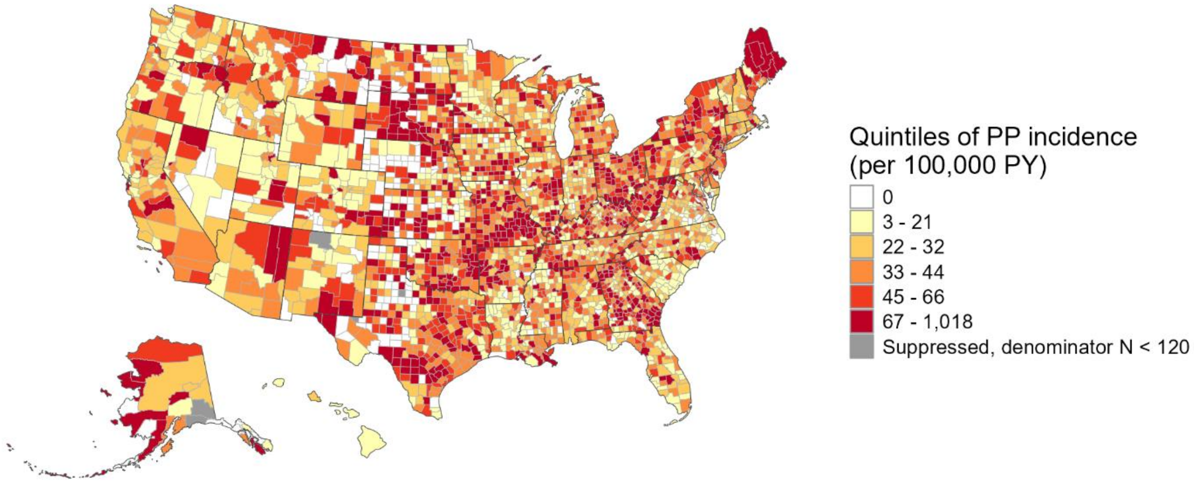

b. Medicaid beneficiaries (2017–2019)

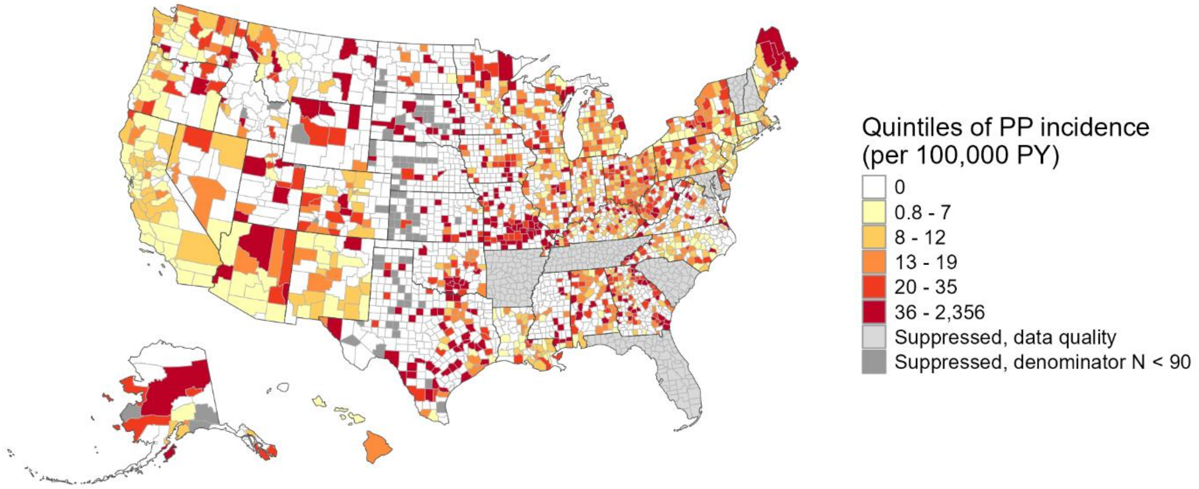

Supplementary Figure 1: Unadjusted county-level PP incidence per 100,000 person-years

The choropleth map shows U.S. counties color-coded based on PP incidence rate quintile.
a. Data for Medicare beneficiaries. b. Data for Medicaid beneficiaries. For Panel A (Medicare), we suppressed data for six counties with < 120 person years. For Panel B (Medicaid), data from Arkansas, Florida, Maryland, New Hampshire, Rhode Island, South Carolina, Tennessee, and Vermont were suppressed due to data quality below desired standards. We also suppressed data for 84 counties with < 90 person years. Very small and very large disease incidence rates are plausible for counties with small populations. State borders are in black lines and county borders are in gray.

FFS = fee-for-service; MA = Medicare Advantage; PP = pneumococcal pneumonia PY = person-years.

a. Medicare FFS and MA beneficiaries

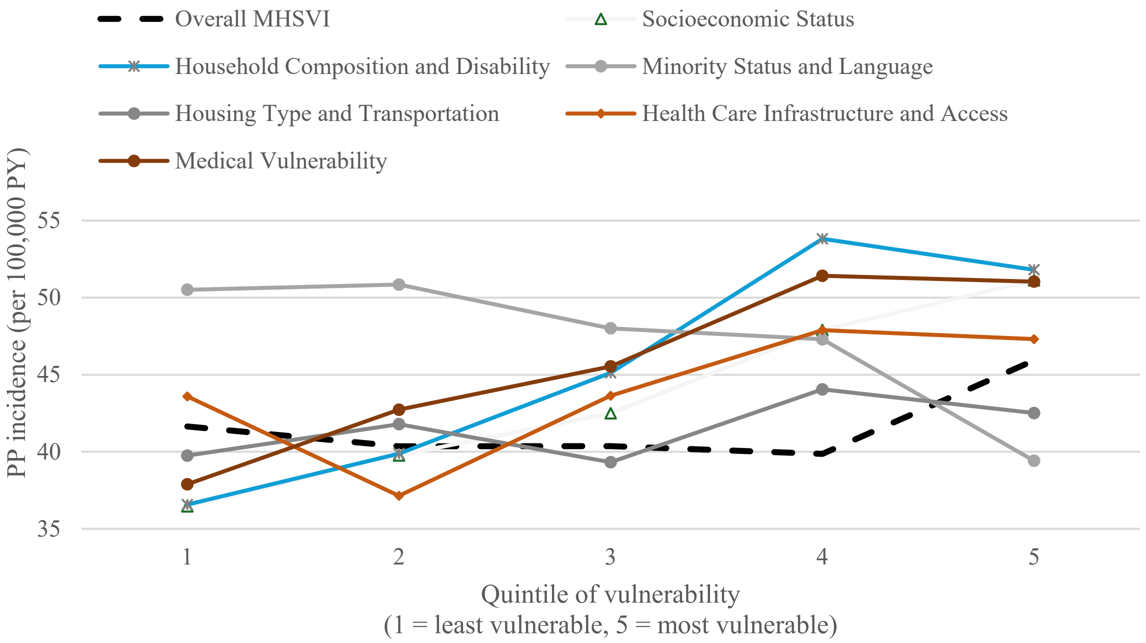

b. Medicaid beneficiaries

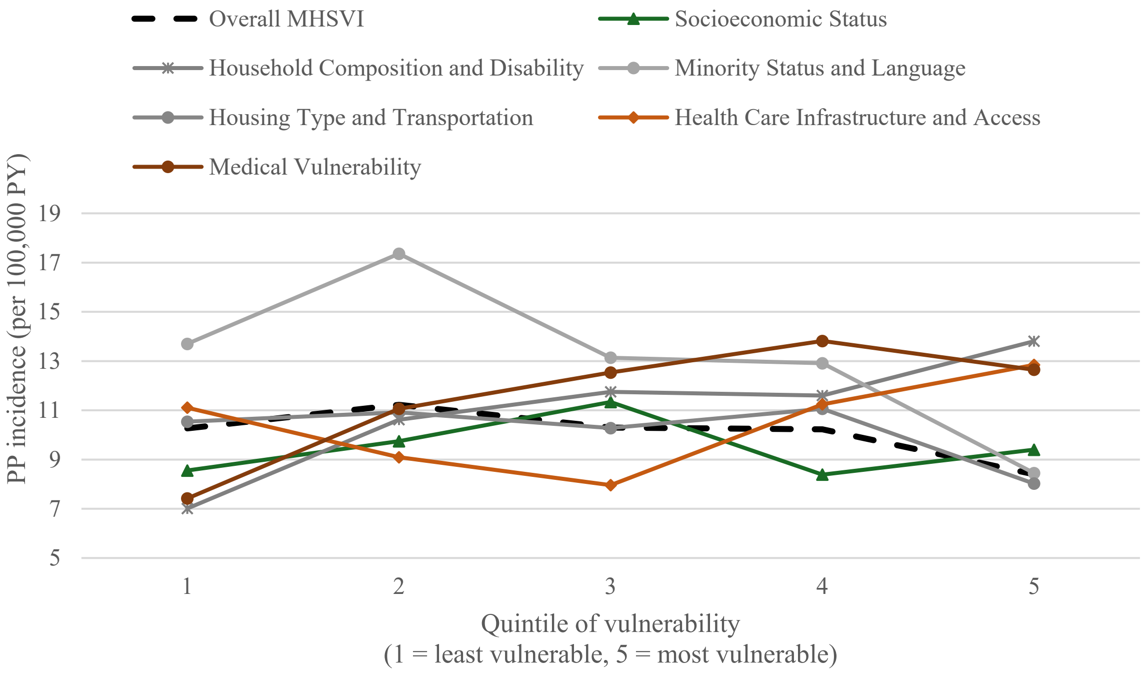

Supplementary Figure 2: Unadjusted PP incidence per 100,000 person-years by quintile of MHSVI and its themes

a. Data for Medicare and FFS beneficiaries. b. Data for Medicaid beneficiaries. FFS = fee-for-service; MA = Medicare Advantage; MHSVI = Minority Health Social Vulnerability Index; PP = pneumococcal pneumonia; PY = person-years.

a. Medicare FFS and MA beneficiaries (2016–2019)

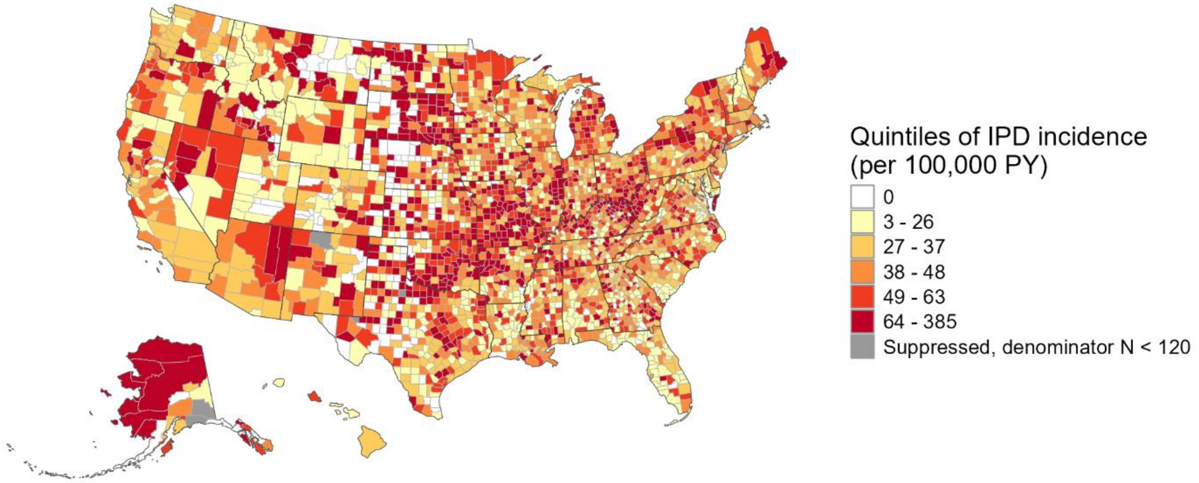

b. Medicaid beneficiaries (2017–2019)

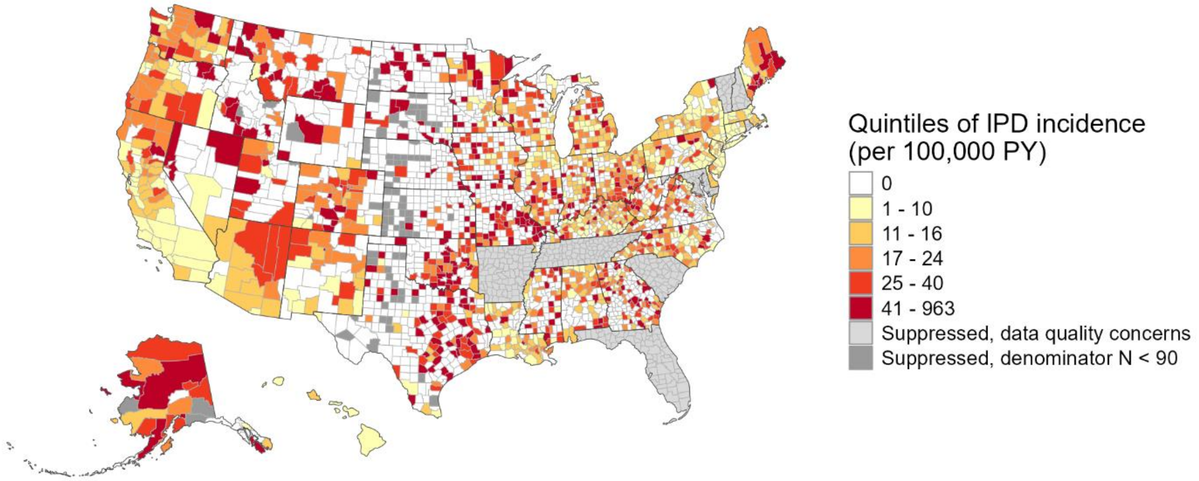

Supplementary Figure 3: Unadjusted county-level IPD incidence per 100,000 person-years

The choropleth map shows U.S. counties color-coded based on IPD incidence rate quintile. For Panel A (Medicare), we suppressed data for six counties with < 120 person years. For Panel B (Medicaid), data from Arkansas, Florida, Maryland, New Hampshire, Rhode Island, South Carolina, Tennessee, and Vermont were suppressed due to data quality issues. We also suppressed data for 84 counties with < 90 person years. Very small and very large disease incidence rates are plausible for counties with small populations. State borders are in black lines and county borders are in gray.

FFS = fee-for-service; IPD = invasive pneumococcal disease; MA = Medicare Advantage; PY = person-years.

a. Medicare FFS and MA beneficiaries

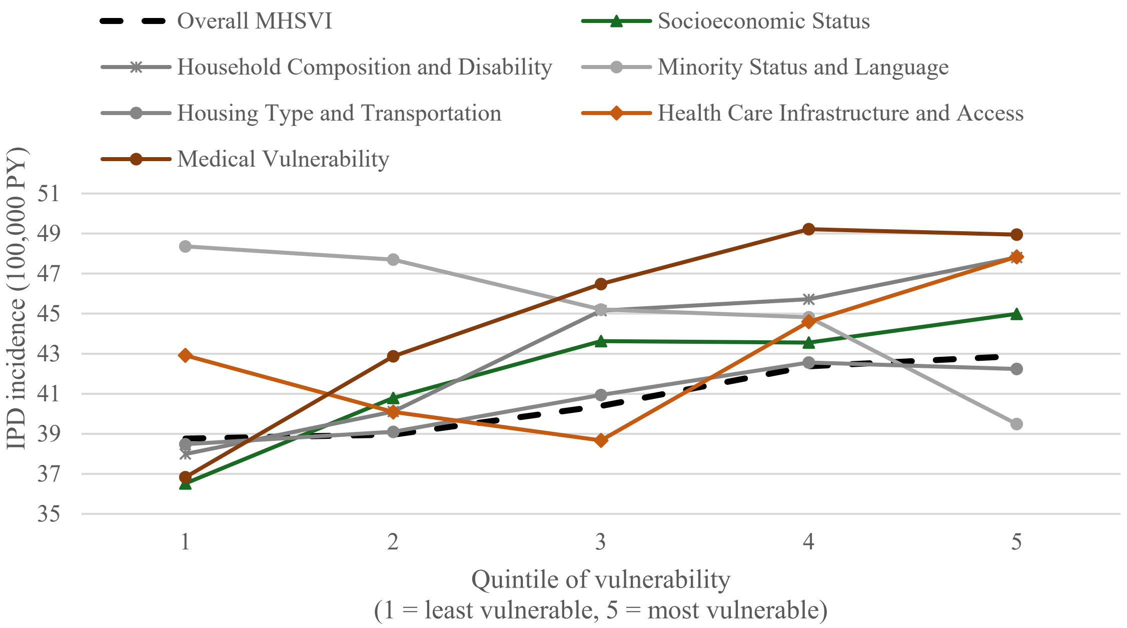

b. Medicaid beneficiaries

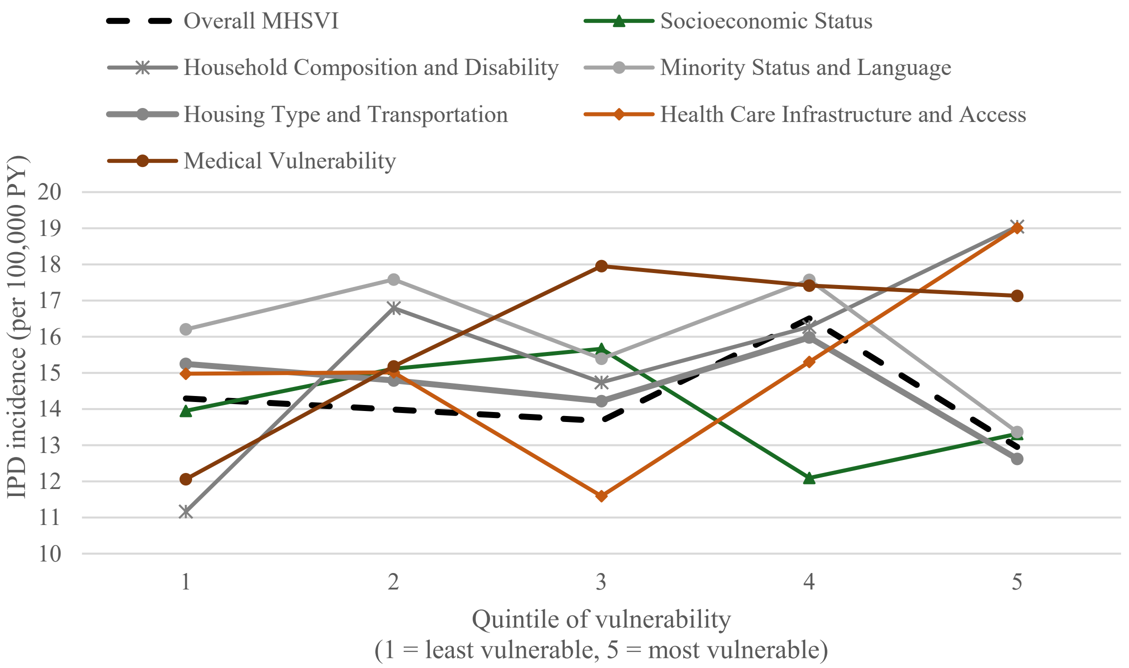

Supplementary Figure 4: Unadjusted IPD incidence per 100,000 person-years by quintile of MHSVI and its themes

a. Data for Medicare and FFS beneficiaries. b. Data for Medicaid beneficiaries. FFS = fee-for-service; IPD = invasive pneumococcal disease; MA = Medicare Advantage; MHSVI = Minority Health Social Vulnerability Index; PY = person-years.
