# Supplementary material for: Influence of area-level social vulnerability on all-cause pneumonia incidence among adult Medicare and Medicaid enrollees
Source: Commun Med (Lond). 2025 Nov 14;5:467. doi: 10.1038/s43856-025-01163-4 (PMC12618641; doi:10.1038/s43856-025-01163-4)
Supplement: Supplementary file 2 — Description of Additional Supplementary Files [file 43856_2025_1163_MOESM2_ESM.docx]

**Description of Additional Supplementary Files**

File name: Supplementary Data 1

Description: Underlying data for Figure 1: unadjusted county-level ACP incidence among Medicare enrollees (2016-2019)

File name: Supplementary Data 2

Description: Underlying data for Figure 2: unadjusted ACP incidence among Medicare enrollees by overall MHSVI and theme quintile (2016-2019)

File name: Supplementary Data 3

Description: Underlying data for Figure 3: unadjusted county-level ACP incidence among Medicaid enrollees (2017-2019)

File name: Supplementary Data 4

Description: Underlying data for Figure 4: unadjusted ACP incidence by MHSVI overall and theme quintiles among Medicaid enrollees (2017–2019)

File name: Supplementary Data 5

Description: Table of diagnosis codes used to identify ACP, PP, and IPD

File name: Supplementary Data 6

Description: Table of diagnosis and procedure codes used for moderate risk classification

File name: Supplementary Data 7

Description: Table of diagnosis and procedure codes used for high risk classification
