# Supplementary material for: Influence of area-level social vulnerability on all-cause pneumonia incidence among adult Medicare and Medicaid enrollees
Source: Commun Med (Lond). 2025 Nov 14;5:467. doi: 10.1038/s43856-025-01163-4 (PMC12618641; doi:10.1038/s43856-025-01163-4)
Supplement: Supplementary file 10 — Reporting Summary [file 43856_2025_1163_MOESM10_ESM.pdf]

nature portfolio

Corresponding author(s): Salini Mohanty, salini.mohanty@merck.com

Last updated by author(s): Jul 11, 2025

Reporting Summary

Nature Portfolio wishes to improve the reproducibility of the work that we publish. This form provides structure for consistency and transparency in reporting. For further information on Nature Portfolio policies, see our Editorial Policies and the Editorial Policy Checklist.

Statistics

For all statistical analyses, confirm that the following items are present in the figure legend, table legend, main text, or Methods section.

n/a	Confirmed	
☐	☒	The exact sample size (*n*) for each experimental group/condition, given as a discrete number and unit of measurement
☐	☒	A statement on whether measurements were taken from distinct samples or whether the same sample was measured repeatedly
☐	☒	The statistical test(s) used AND whether they are one- or two-sided<br>*Only common tests should be described solely by name; describe more complex techniques in the Methods section.*
☐	☒	A description of all covariates tested
☐	☒	A description of any assumptions or corrections, such as tests of normality and adjustment for multiple comparisons
☐	☒	A full description of the statistical parameters including central tendency (e.g. means) or other basic estimates (e.g. regression coefficient) AND variation (e.g. standard deviation) or associated estimates of uncertainty (e.g. confidence intervals)
☐	☒	For null hypothesis testing, the test statistic (e.g. *F*, *t*, *r*) with confidence intervals, effect sizes, degrees of freedom and *P* value noted<br>*Give P values as exact values whenever suitable.*
☒	☐	For Bayesian analysis, information on the choice of priors and Markov chain Monte Carlo settings
☒	☐	For hierarchical and complex designs, identification of the appropriate level for tests and full reporting of outcomes
☐	☒	Estimates of effect sizes (e.g. Cohen's *d*, Pearson's *r*), indicating how they were calculated

Our web collection on statistics for biologists contains articles on many of the points above.

Software and code

Policy information about availability of computer code

Data collection	No software was used for data collection. We used secondary data.
Data analysis	The code used to analyze the data and generate findings of this study cannot be made publicly available, due to proprietary restrictions and data use agreement requirements. Analyses were conducted using SAS version 9.3 or later and R version 4.4.0.

For manuscripts utilizing custom algorithms or software that are central to the research but not yet described in published literature, software must be made available to editors and reviewers. We strongly encourage code deposition in a community repository (e.g. GitHub). See the Nature Portfolio guidelines for submitting code & software for further information.

Data

Policy information about availability of data

All manuscripts must include a data availability statement. This statement should provide the following information, where applicable:

- Accession codes, unique identifiers, or web links for publicly available datasets
- A description of any restrictions on data availability
- For clinical datasets or third party data, please ensure that the statement adheres to our policy

The data of Medicare and Medicaid beneficiaries are available under restricted access due to the requirements from CMS. Our data use agreement (DUA) prevents us from sharing data so cannot be made publicly available. According to the DUA, our group can only access data through the Virtual Data Resource Center (VRDC) with controlled access and cannot download the data. MHSVI data are available as of February 25, 2025 at https://minorityhealth.hhs.gov/minority-health-svi.

Research involving human participants, their data, or biological material

Policy information about studies with human participants or human data. See also policy information about sex, gender (identity/presentation), and sexual orientation and race, ethnicity and racism.

Reporting on sex and gender	Enrollee sex (male, female) as defined in Medicare and Medicaid enrollment data was used as a covariate and subgroup. Medicaid enrollees missing information on sex (n=2,000) were excluded from the models.<br><br>Of the 56,909,851 Medicare enrollees analyzed, 31,307,787 were female.<br><br>Of the 36,579,701 Medicaid enrollees analyzed, 21,319,969 were female.
Reporting on race, ethnicity, or other socially relevant groupings	We used the Minority Health Social Vulnerability Index (MHSVI), a composite score released by the CDC (Office of Minority Health 2023) to characterize county-level social vulnerability across all 50 U.S. states and the District of Columbia. The MHSVI we used was based on 2018 data and was made available in 2021. The index includes six themes: Socioeconomic Status, Household Composition and Disability, Minority Status and Language, Housing Type and Transportation, Health Care Infrastructure and Access, and Medical Vulnerability (Table 1). The composite and theme indices are scored from 0 to 1, with higher values indicating higher social vulnerability.<br><br>By grouping individuals according to the vulnerability level of their county of residence, we assess whether living in a more versus less vulnerable area is associated with different disease incidence—even after accounting for individual demographic and clinical characteristics. The underlying premise is that area-level conditions can shape individuals' health risks through structural and environmental pathways. For example, Housing Type and Transportation vulnerability may increase individuals' exposure to infection through crowded living conditions or limit access to healthcare due to lack of transportation. Healthcare Infrastructure and Access reflects the density of local providers and facilities, which influences how easily individuals can obtain care. However, we do not hypothesize or test for specific directional effects as our analysis framework is interpretive, rather than causal.<br><br>To evaluate how incidence rates (for each outcome and demographic subgroup) varied by area-level social vulnerability (based on the overall MHSVI index and each of the six theme indices), we assigned counties an MHSVI quintile, which ranged from 1 as least vulnerable ($0 \leq$ MHSVI < 0.2) to 5 as most vulnerable ($0.8 \leq$ MHSVI ≤ 1). We then calculated disease incidence by MHSVI quintile by aggregating enrollee-level data for enrollees residing in counties assigned to each MHSVI quintile, based on enrollees' most recent county of residence. We used a paired t-test to assess whether the incidence rate ratio (IRR), reflecting the ratio of disease incidence between counties in the fifth versus first MHSVI quintiles (Q5/Q1), differed significantly.<br><br>Lastly, we used Poisson regression to evaluate the relationship between area-level vulnerability and disease incidence, controlling for potential confounding by individual-level characteristics. We fit separate models for the Medicare and Medicaid cohorts. Our outcome was the count of disease episodes an enrollee experienced during the study period, with an offset term that captures the number of person-years each enrollee contributed during the study period. Our unadjusted models included only indicators for the MHSVI quintiles, whereas adjusted regression models included covariates. These covariates included age group (for Medicaid: 19–49, 50–64 years; for Medicare: 65–74, 75–84, 85+ years), dual enrollment in Medicare and Medicaid (yes, no; controlled for in Medicare cohort models only), sex (male, female; Medicaid enrollees missing information on sex [n=2000] were excluded from the models), pneumococcal disease risk status (low risk, moderate risk, high risk), race and ethnicity (non-Hispanic White, non-Hispanic Black, Hispanic, Asian, Other, missing), and urbanicity (urban, suburban, rural). We defined urbanicity using the 2013 Rural-Urban Continuum Codes developed by the U.S. Department of Agriculture, considering all metropolitan counties as urban, all non-metropolitan counties with 2,500 people or more as suburban, and counties with fewer than 2,500 people as rural.<br><br>To assess the influence of potential confounders on the extent to which area-level vulnerability predicted disease incidence, we examined how the difference in disease incidence between MHSVI Q5 and MHSVI Q1 changed between the unadjusted and adjusted regression models.
Population characteristics	Of the 56.9 million Medicare enrollees analyzed, most were female (55%), non-Hispanic White (81%), 65 to 74 years of age (51%), at high risk for pneumococcal disease (51%), and lived in an urban area (83%). See Table 2 for more information.<br><br>Of the 36.6 million Medicaid enrollees analyzed, most were female (58%), 19 to 49 years of age (80%), at low risk for pneumococcal disease (77%), and lived in an urban area (86%). See Table 4 for more information.
Recruitment	Not applicable.
Ethics oversight	This study did not require patient institutional review board (IRB) or ethics committee (EC) review. Based on restrictions placed through the DUA with CMS, we are granted approval for studies of population health and of access to care, such as this study.

Note that full information on the approval of the study protocol must also be provided in the manuscript.

Field-specific reporting

Please select the one below that is the best fit for your research. If you are not sure, read the appropriate sections before making your selection.

☒ Life sciences ☐ Behavioural & social sciences ☐ Ecological, evolutionary & environmental sciences

For a reference copy of the document with all sections, see nature.com/documents/nr-reporting-summary-flat.pdf

Life sciences study design

All studies must disclose on these points even when the disclosure is negative.

Sample size

Our study population includes two cohorts: (1) adults ages 65+ years enrolled in Medicare fee-for-service or Medicare Advantage plans, and (2) adults ages 19-64 years enrolled in Medicaid. These nationwide samples were sufficient for our analysis, even after applying exclusions detailed below.

Data exclusions

To be included in the analyses, Medicare enrollees had to be continuously enrolled for at least one year during the study period. Inclusion criteria for Medicaid enrollees required continuous enrollment for at least six months during the study period, residence in a state with reliable claims data, and residence in a county with available MHSVI data. We required a shorter continuous enrollment for Medicaid because Medicaid eligibility is determined by several criteria that change over time and vary by state, whereas with Medicare, most of the population includes individuals who are permanently eligible starting at age 65. These enrollment continuity and length restrictions were applied to avoid potential bias, whereby enrollees could have had fewer observable disease events because of enrollment gaps or a shorter length of enrollment. We excluded from the Medicaid cohort enrollees who were dually enrolled in Medicare and Medicaid because Medicare is the primary payer and we cannot capture disease episodes reliably in Medicaid alone. We also excluded Medicaid enrollees receiving long-term care services during the study period, since claims do not reliably capture procedure codes, which are used to classify risk of pneumococcal disease.

Replication

Not relevant: data included was population-wide, among those eligible for inclusion in the study.

Randomization

Not relevant: retrospective cohort study.

Blinding

Not relevant: retrospective cohort study.

Reporting for specific materials, systems and methods

We require information from authors about some types of materials, experimental systems and methods used in many studies. Here, indicate whether each material, system or method listed is relevant to your study. If you are not sure if a list item applies to your research, read the appropriate section before selecting a response.

Materials & experimental systems

n/a	Involved in the study	
☒	☐	Antibodies
☒	☐	Eukaryotic cell lines
☒	☐	Palaeontology and archaeology
☒	☐	Animals and other organisms
☒	☐	Clinical data
☒	☐	Dual use research of concern
☒	☐	Plants

Methods

n/a	Involved in the study	
☒	☐	ChIP-seq
☒	☐	Flow cytometry
☒	☐	MRI-based neuroimaging

Plants

Seed stocks

Not applicable

Novel plant genotypes

Not applicable

Authentication

Not applicable
